# Integrated human toxicokinetics of acetamiprid using urine, blood, and feces data in physiologically-based kinetic modelling for reverse dosimetry

**DOI:** 10.1007/s00204-026-04459-z

**Published:** 2026-05-25

**Authors:** N. C. Wieland, A. Noorlander, A. Zwartsen, R. Greupink, M.H.F. Graumans, H. Mol, J. Dias, F. G. M. Russel, A. M. J. Ragas, P. T. J. Scheepers

**Affiliations:** 1 Department of Environmental Science, Radboud Institute for Biological and Environmental Sciences, Radboud University, Nijmegen, The Netherlands; 2 Wageningen Food Safety Research, Wageningen University and Research, Wageningen, The Netherlands; 3 National Institute for Public Health and the Environment (RIVM), 3721 MA Bilthoven, The Netherlands; 4 Department of Pharmacy, Pharmacology and Toxicology, Radboud University Medical Center, Nijmegen, The Netherlands

**Keywords:** Computational modelling, Pesticide exposure, Risk assessment, Neonicotinoids, Volunteer

## Abstract

Acetamiprid (ACE) is a widely used neonicotinoid insecticide with known neurotoxic potential, for which the European Food Safety Authority lowered the acceptable daily intake. Current physiologically-based kinetic (PBK) models lack adequate kinetic data for ACE and its metabolite acetamiprid-N-desmethyl (ACE-DEM). The aim of this study was to generate detailed disposition data for ACE in a controlled volunteer setting to develop a robust PBK model suitable for reverse dosimetry. Four volunteers received an oral dose of ACE (90% of the acceptable daily intake, ADI) and a single dermal administration on a separate occasion. Concentration–time profiles of ACE and ACE-DEM were collected for urine, blood, and feces. A PBK model was developed using toxicokinetic parameters from the volunteer study. The model was validated using previously published human data. Sensitivity analyses identified key parameters of model performance. ACE was rapidly absorbed and extensively metabolized to ACE-DEM. ACE was undetectable in urine after 24 h, while ACE-DEM remained quantifiable for 96 h. Urinary excretion accounted for 8–24% of the dose, with < 2% in feces, indicating near to complete absorption. Dermal uptake was approximately 30%. The PBK model adequately described the relationship between external exposure, plasma concentrations, and urinary excretion. Three different reverse dosimetry methods were compared. The estimated oral doses fell within a threefold margin of the administered doses. In conclusion, a human-data-informed PBK model enables reverse dosimetry to estimate oral ACE exposure from urinary biomonitoring data.

**Supplementary Information:**

The online version contains supplementary material available at 10.1007/s00204-026-04459-z.

## Introduction

In 2021, more than 300,000 tons of pesticides were used across Europe, raising concerns about human health risks from pesticide exposure (Eurostat 2025). Humans are exposed to pesticides through diet, dermal contact, and other routes, but the exact impact of this exposure on human health is unknown. One way of assessing internal pesticide exposure is by analysis of urine or blood plasma from spot samples in human biomonitoring (HBM) studies. However, interpretation of spot sample concentrations in terms of risks is challenging.

In a recent study across multiple vegetable farms, acetamiprid (ACE) was identified as the predominant insecticide used as plant-protection product (PPP) in conventional farming and integrative pest management (Mark et al. 2024). ACE is applied as a foliar insecticide for the control of herbivorous insects (EFSA, 2015). Beyond its use as a PPP it also has an authorization as a biocidal active substance (ECHA 2025).

ACE is a neonicotinoid insecticide which works by blocking the nicotinic acetylcholine receptor (nAChR) in insects (EFSA, 2015). A multi-route exposure to ACE has been established for humans The EFSA has detected residues of ACE on various food products, including aubergines, grapefruits, bell peppers and table grapes, identifying the diet as a primary source of ACE exposure (EFSA, 2023). Occupational exposure also occurs, as previous biomonitoring studies found elevated urinary ACE concentrations in greenhouse workers without protective equipment compared to those wearing protective suits, indicating contribution of dermal exposure (Marín et al. 2004). Understanding the kinetics underlying ACE exposure is needed to interpret HBM data.

ACE exhibits affinity for the nAChR in mammals, causing activation and desensitization of the receptor. This raises concerns about potential human health effects (EFSA, 2024; Phogat et al. 2022). Both ACE and it’s metabolite acetamiprid-N-desmethyl (ACE-DEM) possess the ability to bind to nAChRs (EFSA, 2024). Detection of ACE and ACE-DEM in brain tissue and cerebrospinal fluid of mice and humans indicates that the compounds can affect the nervous system (Ford & Casida 2006; Laubscher et al. 2022). Additionally, in vitro studies suggest that ACE-induced nAChR activation may functionally affect human neuronal cells at low micromolar concentrations (Loser et al. 2021).

Neonicotinoids, including ACE, are suspected to be able to cross the placental barrier resulting in in utero exposure (Zhang et al. 2022). Disturbance of neurotransmitter signaling during critical windows of brain development, including the prenatal period, may lead to long-term alternations in brain connectivity (Grandjean & Landrigan 2006). This has raised concerns about the developmental neurotoxicity of both ACE and ACE-DEM. As the developing brain is sensitive to exogenous disruption during prenatal and early-life stages, risk assessments prioritize the protection of including pregnant women, infants, children and adolescents, which is considered protective for the general population. (Klaassen 2019). In response, the European Food Safety Authority (EFSA) proposed to lower the ADI from 0.025 mg/kg bw/day to 0.005 mg/kg bw/day based on major uncertainties in the evidence for developmental neurotoxicity of ACE and its metabolite (EFSA, 2024). This reduction reflects concerns about nAChR activation and rapid desensitization, which could lead to adverse developmental outcomes (EFSA, 2024).

Physiologically-based kinetic (PBK) models are able to integrate kinetic data and enable reverse dosimetry, linking urinary or blood plasma concentrations measured in biomonitoring studies to external exposure doses (Clewell et al. 2008). This approach allows comparison of calculated exposures to regulatory thresholds such as the ADI or acute reference dose (ARfD), thereby assessing population-level compliance with health-protective limits.

Some information on the kinetics of ACE is available. Following oral exposure in rats, ACE is well absorbed, extensively metabolized, and fully excreted within 24 h, predominantly as the demethylated metabolite acetamiprid-N-desmethyl (ACE-DEM) (EFSA, 2015). A similar conversion of ACE to ACE-DEM is observed in humans (Wrobel et al. 2022). Moreover, in humans ACE metabolism also produces 6-chloronicotinic acid (6-CNA), although this metabolite is common to other neonicotinoids and therefore not specific for ACE exposure (Wrobel et al. 2023).

An existing PBK-model to predict the risk of developmental neurotoxicity after ACE exposure was developed by the Organisation for Economic Co-operation and Development (OECD) (OECD 2022). Knowledge gaps regarding the currently established model have been addressed by EFSA (European Food Safety Authority, 2024). In short, hepatic clearance parameters were extrapolated from animal data. In vitro studies did not show any formation of ACE-DEM by hepatocytes, which contradicts previous human and animal studies (Khidkhan et al. 2021; Kolanczyk et al. 2020; Wrobel et al. 2022). As a result, parameters for ACE-DEM formation and renal clearance were not supported by data and the renal clearance of ACE was based on the total excretion unchanged. This had a high variability and was collected at a relatively late time point (96 h after exposure), as most of the parent excreted as parent occurs within the first 24 h. Consequently, the predicted internal concentrations of ACE by the OECD model are considered highly uncertain. EFSA conducted an extensive literature search to identify and evaluate new kinetic data for model development in PK-Sim. However, they concluded that the available kinetic data set was too limited to develop a sufficiently robust PBK model for a reliable prediction of external exposure.

Current reverse dosimetry approaches rely on simple equations that use the fraction excreted in urine (F_ue_), typically derived from animal studies (Aylward et al. 2017). This introduces uncertainty due to potential interspecies differences in excretion. Moreover, key aspects of ACE metabolism remain uncharacterized, including the cytochrome P450 (CYP) isoforms responsible for conversion to ACE-DEM (EFSA, 2015; Khidkhan et al. 2021; Wrobel et al. 2022). Consequently, no validated model exists that can reliably convert urinary biomonitoring data to external ACE exposure doses in humans.

The aim of this study was to (i) generate human kinetic data following controlled ACE exposure in human volunteers to characterize its metabolism and excretion, and (ii) develop a robust PBK model informed by these data to enable accurate estimation of ACE exposure from urinary biomonitoring. The resulting model supports more reliable risk assessment for both the general population and occupationally exposed workers, thereby supporting regulatory decision-making and public health protection.

## Material and methods

### Human dosing study

Four human volunteers were exposed to ACE on separate occasions, receiving an oral dose corresponding to 90% of the ADI and a dermal dose of 0.1 mg. The dermal dose was determined based on the maximally predicted absorption using the IHSkinPerm model (Tibaldi et al. 2014). Participant data were stored anonymously and securely using CastorEDC.

#### Recruitment and inclusion criteria

Participants were recruited in Nijmegen (The Netherlands) and its surrounding area using printed leaflets, posters and online announcements. Inclusion criteria were good general health (assessed as the absence of health complaints and diagnosed chronic illness), age 18–60 years, and average alcohol consumption of less than two standard glasses a day. Participants who used over-the-counter medication, had skin disorders on the lower forearms or were pregnant or breastfeeding, were excluded.

#### Background exposure and instructions on diet

To limit background exposure, participants were asked to follow a restricted diet consisting of organic fruits and vegetables starting two days before the first dosing (day -2) until the last sampling day (i.e., day 14 after the first dosing). A food diary was kept by participants during this period. In addition, the day prior to and after dermal dosing, participants were asked to refrain from applying skin care products on their lower forearms. During their stay at the Radboud University, participants received food and beverages with no or low probability of ACE residues. Information about other routes of exposure (e.g., working in the garden or applying topical medication to pets) was collected via a questionnaire during the first visit (see Supplementary S1 for the questionnaire).

#### Dosing

Four participants (aged 19–57 years) were enrolled in the study between December 2023 and July 2024, including one female and three males. An overview of participant characteristics and their doses can be found in Table 1.

**Table 1:** Participant demographics and administered doses

Volunteers	Age	Weight (kg)	Oral dose (mg)	Dermal dose (mg)
M1 M2 M3 F4	19 57 26 23	59 98 63 85	1.3 2.2 1.4 1.9	0.1 0.1 0.1 0.1

M, male; F, female.

Participants were dosed once orally and once dermally, on two separate occasions. They received a dose corresponding to 90% of the ADI (0.025 mg/kg bw), corrected for their bodyweight. Both doses were prepared in a clean room using analytical standards. Participants were dosed in the early morning (8 a.m.) and asked to abstain from having breakfast unless they could eat more than 2 h before. For oral administration participants were instructed to drink the solution in less than one minute. On a separate occasion a solution of ACE in acetone was applied using a pipette to approximately 25 cm^2^ of the skin of the lower forearm and left uncovered to allow the solvent to evaporate. After 4 h, the skin was wiped using a ghost wipe (SKC Ltd., Dorset, UK) and washed by the participant with soap to ensure no residue was left on the skin.

#### Sample collection

A visual illustration of sample collection can be found in Supplementary S2. After oral or dermal exposure, participants were instructed to collect 24-h urine in separate containers (per void) and to collect the first morning voids after awakening until 96 h after dosing started. Whole fecal voids were collected using Fecotainers (Excretas Medical, Enschede, the Netherlands) for the whole study duration. Urine and fecal samples collected at home were kept in an electronically-powered cool-box with cool packs provided by the researcher until delivery at the Radboud University where samples were frozen (− 18 °C). A baseline blood sample was drawn at the start of both dosing scenarios, 30 min before dosing. After dosing either orally or dermally, venous blood was collected in plasma separation tubes (PST II, BD) for a total period of 10 h using an intravenous cannula (Supplementary S2). Blood samples were then centrifuged (10 min, 2500 RCF) and 1 mL of plasma was aliquoted. The aliquoted samples were stored at − 20 °C until laboratory analysis. On the mornings after dosing, a single blood sample was collected with a butterfly needle up to 96 h post-dosing. One day before blood plasma extraction the aliquoted samples were stored in the fridge at 4–8 °C. Urine samples were aliquoted and stored at − 18 °C until transport within 48 h after collection. The urine samples were transported frozen on dry ice to Wageningen Food Safety Research (WFSR) and stored at − 80 °C until analysis. Fecal samples were also transported frozen on dry ice and stored at − 18 °C until analysis.

### Laboratory analysis

#### Reference standards

ACE, ACE-DEM, Chloridazon-D5, Bentazone-D6, Diuron-D6 and 2,4-D 13-D6 standards were obtained from LGC Standards (Dr Ehrenstorfer, > 99% purity). For dosing, ethanol with 96% purity was used to dissolve the analytical standard at a concentration of 1 mg/mL. The stock was stored at 8° Celsius and used to obtain dilutions at each day of the dosing study. The stock was diluted using tap water, resulting in a final ethanol concentration of 0.1% and a dose corresponding to 90% of the ADI for ACE.

For dermal application, ACE was applied at a dose of 0.1 mg for all participants. Preparations were made in acetone, as acetone evaporates and leaves the dissolved compound as a residue on the skin. This methodology is based on a previous controlled exposure study (Oerlemans et al. 2019).

Other chemicals needed for the preparation of extraction, buffer and eluent solutions were all of analytical purity or higher. Ultrapure water had a resistivity of 18.2 Ω•cm and was freshly tapped using an Elga LabWater system (Veolia, Netherlands). Used chemicals were ammonium formate (NH_4_HCO_2_), magnesium sulfate (MgSO_4_), sodium acetate (CH_3_COONa), dimethyl sulfoxide (DMSO) formic acid (FA), acetonitrile (ACN) and methanol (MeOH) were purchased at Merck (Amsterdam, the Netherlands).

#### LC–MS/MS analysis

Blood plasma, urine and feces were analyzed based on earlier published methodology (dos Santos et al. 2024). Briefly, blood plasma samples were aliquoted and extracted using a quick, easy, cheap, effective, rugged and safe (QuECHERS) method and liquid chromatography tandem mass spectrometry (LC–MS/MS) analysis was performed to quantify ACE and ACE-DEM. A calibration line, including ACE and ACE-DEM, was prepared using pooled human plasma (SEQUENS, VWR, Netherlands). The concentrations of the calibration standards ranged from 0.125 to 10 ng/mL (*n* = 6 calibration points). Pooled human plasma samples were spiked and extracted. The phlebotomy plasma samples were also extracted using the QuECHERS methodology. Subsequently, 1 mL of human plasma was spiked with 5 µL of internal standard 1:1 (v/v) mixture containing chloridazon-D5 and bentazone-D6. 3 mL of ACN/0.1% Formic acid (FA) was added and vortexed to precipitate the blood plasma. For additional phase separation, 1.5 g of MgSO_4_/NaAc was added. The mixture was vortexed, centrifuged at 3150 RCF and 1 mL of the ACN layer was taken, spiked with 10 µL of DMSO and subsequently evaporated under a stream of nitrogen until near dryness. The final preparation for LC–MS/MS injection included a reconstitution in 100 µL of a solution of 5 mM Ammonium formate (AmFo)/MeOH, 50:50 by volume (corresponding to mobile phases A and B). After centrifuging (5 min, 3150 RCF), 5 µL of internal standard 2 (1:1 (v/v) mixture of Diuron-D6 and 2,4-D 13-C6) was added to the LC–MS/MS vials in combination with reconstituted 80 µL sample. The LC–MS/MS analysis was performed according to the validated methodology which followed Santé guidelines for validation and analytical quality control (SANTE/2020/12830, 2021). A gradient program with two mobile phases was used (Mobile phase A consisted ultrapure water with 5 mM AmFo and spiked with 0.1% formic acid and mobile phase B consisted of 5 mM MeOH spiked with 0.1% formic acid). During ACE standard direct MS/MS infusion, IntelliStart software was used to optimize precursor and daughter ion mass/charge transitions to quantify ACE concentration in the samples. The mass transitions selected for quantification and qualification of ACE were 223 > 126 and 223 > 90 based on prior studies (Harada et al. 2016; Wrobel et al. 2023). For ACE-DEM, the selected mass transitions for quantification and qualification were 211 > 128 and 209 > 126.

For urine analysis, isotopically labeled internal standards (ACE-^13^C_6_, ACE-DEM-^13^C3D) was added to 3 mL urine at 0.5 µg/L. Urine was extracted by adding 6 mL acetonitrile containing 0.1% acetic acid by shaking for 30 min. Then a mixture of magnesium sulfate plus sodium acetate was added (2.4 g + 0.6 g) for phase separation. After shaking and centrifugation, the acetonitrile phase was evaporated and redissolved in 300 µL of methanol/water (10:90) with 0.1% acetic acid. Extracts were analysed by LC–MS/MS (Sciex 6500 + triple quadrupole). The injection volume was 5 µL. For LC a Waters analytical column HSS T3, 1.8 µm, 2.1 × 100 mm was used, and a gradient of water/methanol, containing 5 mM AmFo and 0.1% FA. The LOQ was 0.1 ng/mL for both ACE and ACE-DEM.

For analysis of feces, samples were allowed to reach room temperature. 2 g of sample (wet weight) were aliquoted in an extraction tube. After addition of isotopically labeled internal standards (ACE-^13^C_6_, ACE-DEM-^13^C3D), 4 mL of water and 4 mL of acetonitrile containing 1% of acetic acid were added for extraction (30 min, head-over-head). Then a mixture of magnesium sulfate plus sodium acetate was added (2.4 g + 0.6 g) for phase separation. An aliquot of 250 µL of the acetonitrile phase was diluted with 250 µL of water. The tubes were shaken followed by addition of 1.6 g of magnesium sulfate and 0.4 g of sodium acetate. Extracts were analysed by LC–MS/MS (Sciex 6500 + triple quadrupole). The injection volume was 5 µL. For LC a Waters analytical column HSS T3, 1.8 µm, 2.1 × 100 mm was used, and a gradient of water/methanol, containing 5 mM AmFo and 0.1% FA. The LOQ of was 1 µg/kg for both compounds. More QA/QC data can be found in Supplementary S3**.**

### Physiologically-based kinetic model

#### Model description

**Fig. 1:**
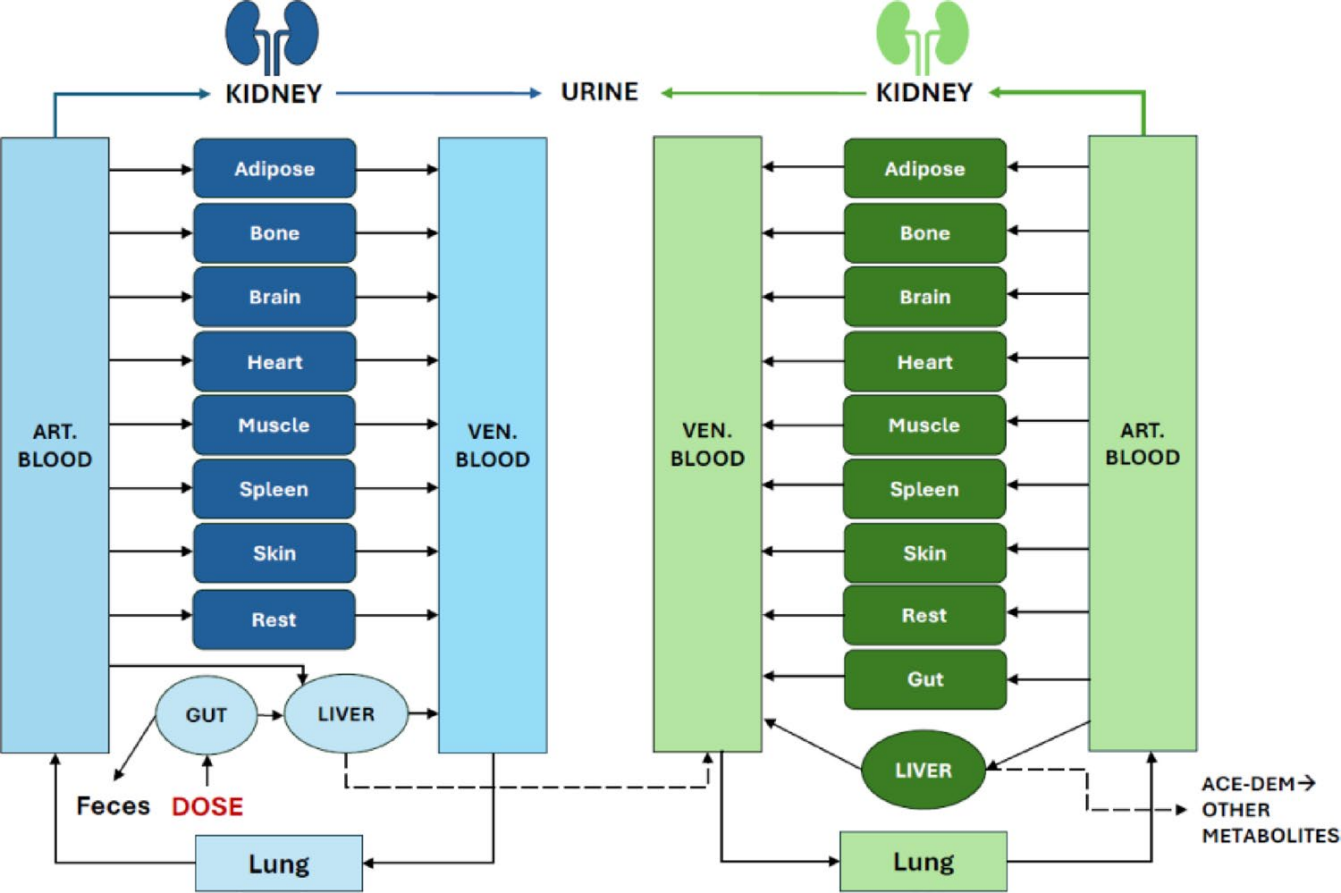
Schematic representation of the PBK model for ACE (left) including an ACE-DEM sub-model (right). After oral exposure, ACE (blue arrows) is absorbed into the systemic circulation via the gut and liver, distributed across physiological compartments, metabolized primarily in the liver to form ACE-DEM (green arrows) and partially excreted via the kidneys into the urine. The metabolite is subsequently distributed and cleared through the kidneys and further metabolized. Solid arrows indicate blood flow; dashed arrows represent biochemical transformation or excretion

A human physiologically-based kinetic (PBK) model was developed to simulate concentration–time profiles of ACE blood plasma levels and urinary ACE-DEM excretion after oral or dermal ACE exposure. In Fig. 1 a schematic representation of the PBK model is presented. In short, the model consists of ordinary differential equations (ODEs) describing the rate of change of mass in fluids and tissues (in mg/h). Differential equations were solved using R package *mrgsolve* (R Core Team 2021).

The model consists of 2 sub-models, 1 describing ACE and 1 describing ACE-DEM. Compartments included in both sub-models are adipose tissue, bone, brain, heart, kidney, liver, lung, muscle, spleen, skin, arterial blood, venous blood, gut (as stomach plus intestine) and a residual compartment, with blood circulation linking them as fraction of the total cardiac output that an organ receives (Brown et al. 1997). Inclusion of these tissues was decided based on potential future application of the model for other pesticides in risk assessment, where the amount of a PPP in each organ can be predicted. All organ tissues were modelled under the assumption of flow-limited (perfusion-limited) kinetics.

#### Model parameterization

##### Absorption

Absorption of ACE from the stomach and skin was modelled using a constant absorption rate (K_a,_ h^−1^), estimated from the slope of the increase in blood plasma concentrations observed between baseline (pre-dose) and C_max_ from the human volunteer study.

Oral bioavailability was assumed to be 98%, based on the absorption characteristics in animal studies (EFSA, 2015). The unabsorbed fraction is calculated from the excreted unchanged amount in the feces. Based on the low levels measured in bile-cannulated rat studies, enterohepatic circulation was not considered a main pathway in the kinetics of ACE (EFSA, 2015).

##### Distribution

The model incorporated the compound-specific parameters logP (1.4 and 1.3 for ACE and ACE-DEM, resp.), pKa (0.7 for both) and molecular weight (222.68 and 208.65 for ACE and ACE-DEM, resp.). The ratio between whole blood and plasma was measured by the OECD, but a description of how this ratio was derived is not available. As such, a standard value of 1 was chosen.

Since no experimental data on partitioning between whole blood and plasma was available, this was assumed to be 1. The previous model obtained a value for the B:P of 0.951, but no description of how this value was derived experimentally was included. Thus, the standard value of 1 was chosen. The unbound fraction for ACE and its metabolite were obtained from Taira et al. (2025). All parameters, and their sources, can be found in Supplementary Table S4.

The tissue/blood partition coefficients were estimated in silico using Rodger’s and Rowlands formula for weak bases, which takes into account the ionization state of the compound (Rodgers & Rowland 2006). Average physiological data on organ volume and blood flows were obtained from the International Commission of Radiological Protection (ICRP, 2002) (12). Organ blood flows were scaled according to the percentage of the cardiac output, while organ volumes were scaled to the bodyweight.

##### Metabolism and excretion

All clearance and metabolic conversion parameters were estimated based on the human volunteer study. Renal clearance (CL_renal_) and metabolic clearance (CL_metabolic_) were estimated for both ACE and ACE-DEM in L/h as described in Supplementary S5. Subscripts denote the compound (ACE or ACE-DEM).

Renal clearance of ACE into the urine was modelled using the parameter CL_renal-ACE_ (L/h). ACE-DEM formed in the liver re-enters the systemic circulation and distributes across all compartments before reaching the kidney for renal clearance, described by CL_renal-ACEDEM_.

Formation of ACE-DEM by CYP450 enzymes was modelled using the metabolic clearance (CL_metabolic, ACE,_ L/h) together with the unbound fraction of ACE in the liver. Further metabolization of ACE-DEM is possible (EFSA, 2024; Harada et al. 2016; Wrobel et al. 2023). To account for this metabolization and the presence of metabolites other than ACE-DEM, the CL_metabolic-ACE-DEM_ for ACE-DEM was fitted based on the urinary excretion data from the human volunteer study.

The urinary excretion rate (in µg/h) was modelled by multiplying the renal clearance (CL_renal-ACEDEM_) with the concentration in the arterial blood corrected for the unbound fraction. To calculate the urinary concentration (in µg/L), the amount excreted was divided by the urine flow per hour (Q_urine_, in L/h). The amount already excreted was subtracted from the amount present in the bladder by multiplying the urine concentration with the urine flow to leave a net flow of urine.

Half-lives (T_1/2_) of both components in blood plasma and urine were calculated with the PKNCA package using the curve stripping method (Denney 2025). In short, all values below the detection limit and at or before the end of the last dose administration were dropped. Then, the points T_max_ to the last collected sampling point (T_last_) are selected to calculate the T_1/2_.

#### Model evaluation

An external dataset was used to validate the model outputs (Wrobel et al. 2023). In short, their study aimed to quantify the kinetics of multiple neonicotinoids and to identify appropriate biomarkers for each compound. The dataset contained measurements of ACE and ACE-DEM in urine over 48 h after a single dose of ACE based on the ADI. The doses ranged from 0.012 to 0.060 mg/kg bw. Three male participants were included (50 years, 83 kg; 37 years, 98 kg; 48 years, 96 kg). These data were used to validate the modelled urine concentrations after calibration. In this study no blood plasma or feces were collected.

#### Sensitivity analysis

(Loizou et al. 2015; McNally et al. 2011)The goal was to develop a model to perform a reverse dosimetry using biomonitoring data. Spot urine is most often collected in biomonitoring research as it is non-invasive and easy to collect. The main outcome of interest was the area under the curve (AUC) of the concentration–time curves for ACE-DEM, which equals the total amount excreted over 5 days. As urine concentrations will be used in the reverse dosimetry calculations, it was important to investigate which parameters had the strongest effect on the AUC of the ACE-DEM concentration–time profiles. A global sensitivity analysis with Morris’ test and EFAST was used based on previously published methods (Loizou et al. 2015; McNally et al. 2011).

(McNally et al. 2011)For Morris’ test, a unified distribution was assumed for all parameters with a deviation of 10%. The test was run with 100 iterations and 6 levels, which is within the recommended 50–200 iterations and 4–10 levels (McNally et al. 2011). Next, for the eFAST analysis, a lognormal distribution was selected for the physiological parameters (bodyweight, cardiac output), and unified for the others with the same 10% deviation as the Morris’ test. A full description of the inputs and outcomes from the sensitivity analysis can be found in Supplementary S6.

Visualization of concentration–time data was done using Rstudio and *ggplot2* packages (R Core Team 2021). The sensitivity analysis was performed using the *sensitivity* package.

#### Reverse dosimetry

After validation, the model was applied to estimate the oral ACE exposure dose based on urinary ACE-DEM using data from the Wrobel et al. study (Wrobel et al. 2023).

The participants from the Wrobel et al. study did not follow an organic diet to restrict background exposure. Baseline samples contained levels of ACE-DEM. Background exposure in the Wrobel et al. participants was probable. ACE and ACE-DEM are often detected in biological media because diet is expected to be the main source of exposure for the general population. Since the diet was not controlled, and concentrations were much higher compared to those in our study, we assumed the exposure of participants in the Wrobel et al. was due to regular ACE intake via the food, and thus at steady state.

Before performing the reverse dosimetry using the PBK-model the modelled urine concentrations at T = 0 needed to match the background levels observed in the Wrobel study. A daily exposure of 25% of the ADI was assumed and modelled for 21 days.

Next, the administered dose, bodyweight and urinary flow were adjusted to match the characteristics of the Wrobel participants. Because in this study the doses, bodyweight and average urine flow were higher than the standard values used during model development, these parameters were expected to vary across a population and can be adjusted to reflect the study design of Wrobel and co-workers. After making these adjustments, reverse dosimetry was performed. Three different methods were applied.

Method 1 is based on the use of the fraction excreted in urine (F_ue_) 24 h after exposure, an approach that has been described previously (Aylward et al. 2017; Wrobel et al. 2023). The F_ue_ was derived from cumulative urinary outputs from the PBK-model and was independent from dose. The estimated dose (ED) was calculated as:

1 $$ED= \frac{{C}_{24} x {V}_{24}}{{F}_{ue}}$$

where *C*_*24*_ is the urine concentration in µg/L in the morning void, the 24 h urine volume (*V*_*24*_) a set value of 1.7 L, and the *F*_*ue*_ equals 0.12.

In method 2 the PBK model is used to establish a linear relationship between urinary concentration and dose. The PBK-model was used to simulate concentration–time curves for 100 doses ranging between 0.1 and 3000 µg. From this, a linear relationship was derived between the oral dose and modelled metabolite urine concentration 24 h after dose:

2 $$ED={C}_{24}*x+b$$

where *C*_*24*_ is the concentration in urine in µg/L 24 h after dosing, *x* is the slope and *b* is the intersection at y.

Method 3 uses the cumulative urinary excretion at 24 h post-exposure. Like method 2, a linear relationship between the cumulative amount excreted in urine at 24 h post-exposure and oral dose was derived:

3 $$ED={A}_{24}*x+b$$

where *A*_*24*_ is the excreted dose in urine in µg/L in 24 h.

EDs were compared to the administered dose. Across the different methods, the % deviation from the administered dose was calculated.

## Results

### Experimental oral exposure

**Fig. 2:**
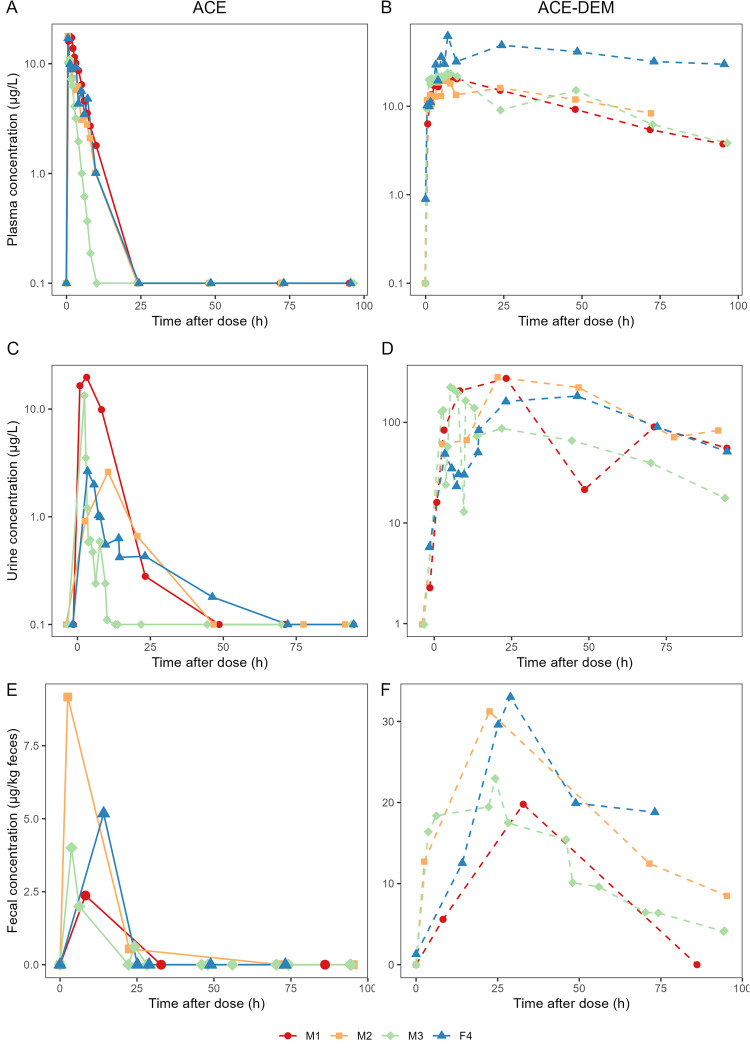
Kinetic profiles of ACE (left column; solid lines) and ACE-DEM (right column; dashed lines) after oral dosing at 90% of the ADI in four human volunteers: 3 females (F) and 1 male (M). Figures show plasma (a-b), urine (c-d) and fecal (e-f) concentration–time profiles (ng/mL) measured over 96 h after the start of exposure. Concentration in feces is measured in wet weight

The measured urine and plasma concentration–time profiles for ACE and ACE-DEM over a 96 h following a single oral dose and the calculated cumulative excretion as a % of the oral dose in urine (Fig. 2). Overall, elimination of ACE was rapid, while the urinary concentration–time profile showed prolonged excretion of ACE-DEM. This finding was supported by the plasma-concentration time profiles, which show the presence of ACE-DEM in blood up until 96 h after dosing. All calculated kinetic parameters for blood plasma can be found in Table 2**.** The oral absorption observed in the study was assumed to be unaffected by factors such as prior food intake or gastric emptying, as participants fasted in the morning before dosing or had breakfast min. 2 h or longer before receiving the dose of ACE.

**Table 2:** Kinetic parameters derived from blood plasma for ACE and ACE-DEM per volunteer

Participant	K_a_	ACE				ACE-DEM				
		T_max_ plasma (h)	C_max_ plasma (µg/L)	T_1/2_ plasma (h)	T_1/2_ urine (h)		T_max_ plasma (h)	C_max_ plasma (µg/L)	T_1/2_ plasma (h)	T_1/2_ urine (h)
M1	15	1.2	18	2.6	2.9		10	20	35	NA*
M2	41	0.4	18	1.9	NA*		7	20	51	29
M3	10	1.1	11	1.2	1.1		7	24	24	26
F4	34	0.5	17	3.0	22		7	62	94	28
Mean (SD)	25 (15)	0.8 (0.4)	16 (3.4)	2.2 (0.8)	8.7 (12)		8.0(1.5)	32 (20)	51 (31)	28 (1.5)

*Not enough points available in the terminal phase to calculate T_1/2_

Ka, absorption coefficient (h−1); Tmax, time to maximum concentration (h); Cmax, maximum concentration (µg/L); T1/2, half life; ACE, acetamiprid; ACE-DEM, Acetamiprid-N-Desmethyl.

**Fig. 3:**
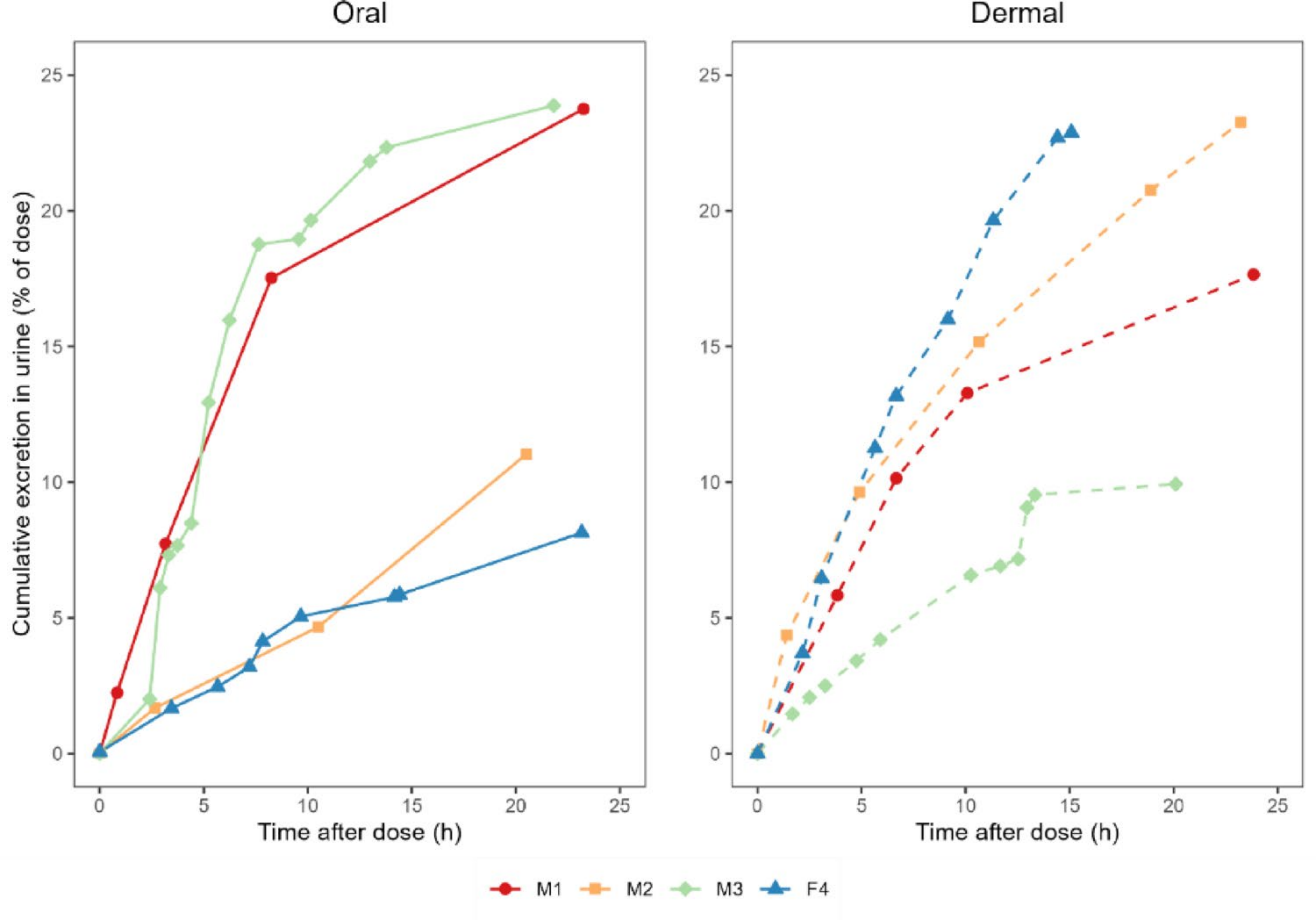
Cumulative excretion in urine as % of dose as ACE (solid line) and ACE-DEM (dashed line) in urine following oral administration at 90% of the ADI or dermal administration at 0.1 mg single dose

Of the oral dose, 8–24% was excreted in urine in 24 h of which 0.2–2.6% as ACE and 7.9–21% as ACE-DEM (Fig. 3). Maximum concentrations of ACE-DEM (C_max_) of 274, 280, 224 and 182 µg/L were reached after 23, 20, 5, and 46 h in urine for M1, M2, M3 and F4, respectively. At the end of the collection period of 5 days, concentrations in morning urine decreased to an average of < LOD for ACE and and 1.9 µg/L for ACE-DEM.

An absorption rate constant of 10 to 41 h^−1^ was calculated. After 24 h, ACE was no longer quantified in urine samples (< LOQ, 0.1 ng/mL), while ACE-DEM was found up to 96 h at concentrations ranging from 17 to 83 µg/L. ACE and ACE-DEM concentrations at baseline were below or close to the LOD (0.01 µg/L). Mean apparent elimination half-life of ACE was 13 h.

The total amount of ACE and ACE-DEM in the fecal voids collected over the study duration revealed a near to complete absorption of ACE from the gut after oral dosing. Baseline samples showed no detectable traces of ACE or ACE-DEM, except in one participant who had a trace level of the metabolite in their baseline fecal sample (1.29 µg/kg; F4). The percentage of the orally administered dose that was recovered after 5 days amounted to a median recovery of < 1% for the parent and 0.7–1.2% for the metabolite, indicating that some of the ingested pesticide is excreted as metabolite in feces. The highest amount in feces was measured 14 h after exposure for the parent and within 24 h for the metabolite. Variation in fecal excretion was within twofold for the T_max_ and threefold for the C_max_ which can possibly be attributed to observed variability in bowel movement patterns between the study participants.

### Experimental dermal exposure

Participants were dermally exposed 7 days after oral exposure. ACE was not detected in the baseline plasma samples of the dermal exposure scenario, but the baseline samples showed concentrations of ACE-DEM between 0.7–7.1 µg/L in plasma, and 3.8–28 µg/L in urine. This is attributed to carry-over resulting from the oral dosing one week prior.

**Fig. 4:**
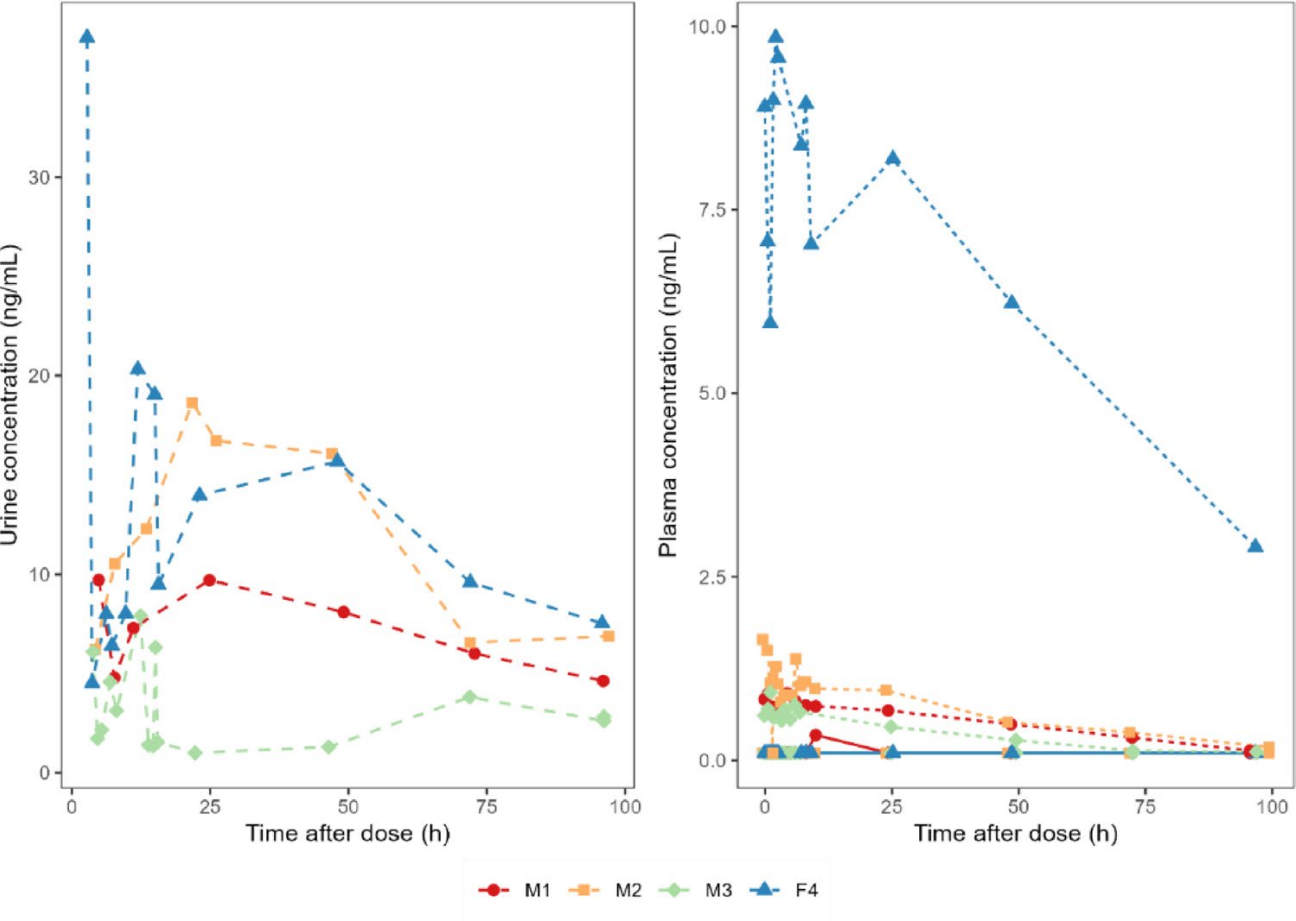
Concentration–time patterns (in ng/mL over 96 h) of ACE (solid line) and ACE-DEM (dashed line) after a dermal dose of 0.1 mg measured in urine (left panel) and plasma (right panel). ACE was below the LOD in urine after dermal exposure, and only in one sample ACE was detected in blood plasma

Concentration–time profiles are summarized in Fig. 4. Minimal skin absorption was observed after dermal dosing. For only one participant, a concentration of 0.3 µg/L ACE was found in blood plasma 10 h post-exposure. For all other participants ACE concentrations remained below LOD in all collected blood samples. No ACE was detected in feces after dermal exposure.

The metabolite was detected in both plasma and urine, with the C_max_ of ACE-DEM being 9.8 µg/L in plasma (T_max_ of 2 h). Of the administered dermal dose of 0.1 mg, between 13 and 30% was excreted in urine within 24 h after exposure. ACE-DEM was detected in feces with a C_max_ of 1.5–5.7 µg/kg feces.

### Sensitivity analyses

Sensitivity analyses were performed to investigate which parameters had the strongest influence on the AUC of the urinary concentration–time curve of ACE-DEM. Morris’ test combined with eFAST showed that the largest effect on the excreted urinary amount of ACE-DEM were attributed to blood:plasma partitioning of ACE (Total sensitivity index (ST) = 0.85, uniform distribution), bodyweight (ST = 0.074, lognormal distribution), CL_metabolic,ACE_ (ST = 0.05), urinary flow (ST = 0.04, uniform distribution), and fraction unbound of ACE in plasma (ST = 0.03, uniform distribution).

### Model calibration

**Fig. 5:**
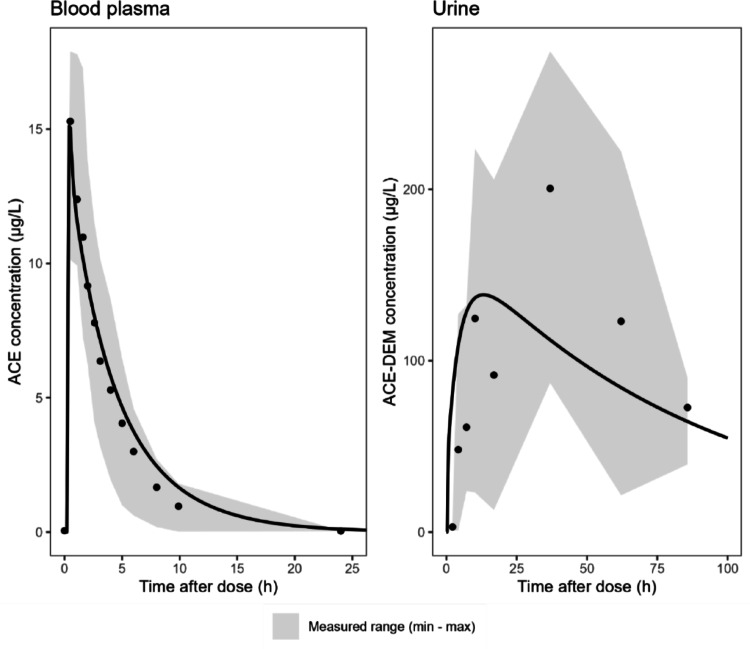
Predicted blood plasma and urinary concentration–time profiles for ACE (left) and ACE-DEM (right) until 96 h after an oral dose of 1.5 mg (90% of the ADI corrected for 70 kg standard bodyweight). Average of measurements from 4 volunteers (dots) and range (grey shaded area)

The model was calibrated to the blood plasma concentrations of ACE using the following approach. First, the influence of the main kinetic parameters identified in the human volunteer study on venous ACE concentrations was evaluated through stepwise adjustment and visual inspection (Supplementary S7). The agreement between measured data and the final calibrated model are shown in Fig. 5**.** The parameters selected for calibration were K_a_, CL_metabolic, ACE_, CL_renal, ACE-DEM_ and CL_metabolic, ACE-DEM_. The K_a_ was reduced from the calculated average of 25 to 15 to improve model fit of the initial absorption phase. This value aligns with K_a_ values on the lower end of the experimentally measured range. This can be justified as the gastrointestinal absorption is easily influenced by factors such as prior food or fluid intake, reflected by the high SD in the measured K_a_. The conversion of ACE to ACE-DEM was underestimated by the initial model, and thus CL_metabolic, ACE_ was scaled from an average of 29 L/h (range 14–54) to 43 L/h. Renal clearance of the metabolite was underestimated, so CL_renal, ACE-DEM_ was scaled from the average of 0.3 L/h (range 0.1–0.6) to 1.2 L/h. CL_metabolic, ACE-DEM_ was increased from the average 0.5 (range 0.06–0.8) to 1.0 L/h. A comparison of the model before and after calibration is included in Supplementary S8.

After calibration, the goodness-of-fit was evaluated using an R^2^ and root mean squared error (RMSE), where a higher R^2^ and a lower RMSE indicate a good fit. For the prediction of ACE blood plasma concentration, an R^2^ of 0.99 and RMSE of 0.525 were calculated. As for the urine concentration, values of -0.041 and 57 were calculated for R^2^ and RMSE respectively.

### Model validation

The model was applied to data from Wrobel et al. (Wrobel et al. 2022), which reported urinary concentrations of 3 volunteers of ACE and ACE-DEM over time following a controlled oral dose. This dataset provided an opportunity to validate simulated concentration–time profiles from our model and the reverse dosimetry-estimated oral dose. A comparison of the from this study, the model predictions and the data from Wrobel et al. is added to the Supplementary data (S9). A few differences can be observed when comparing Wrobel with our study. The samples collected in the Wrobel et al. study showed a background exposure compared to the data collected here. In our study, participants followed an organic diet, where in the Wrobel et al. study volunteers were not asked to restrict their diet. This is reflected in a difference in concentrations over time between the participants from Wrobel et al. and those measured in the current study.

**Fig. 6:**
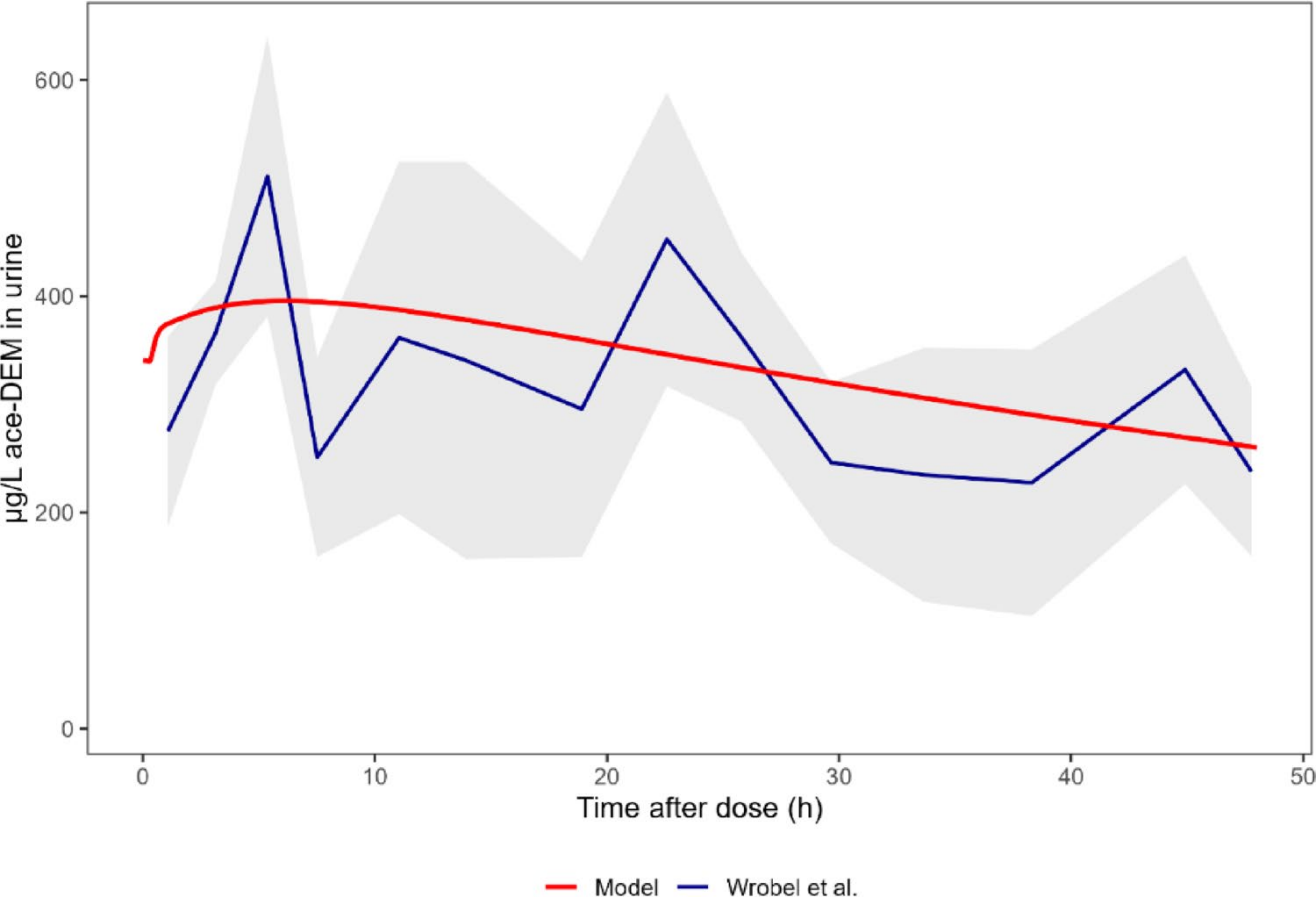
Comparison of the mean (solid line) and range (ribbon) urinary excretion of ACE-DEM (in µg/L) from Wrobel et al. (blue). Model predictions (red) of ACE-DEM in urine (µg/L, at C_ss_, after a single dose of 1.96 mg) compared to measured data from Wrobel et al. (mean, solid blue line; range, shaded). The R^2^ and RMSE were 0.059 and 220, respectively

To better match the conditions of the Wrobel study set-up, the modelled dose was adjusted to the actual oral dose received by the volunteers of the Wrobel study and background exposure was modelled at steady-state to match the baseline urinary concentrations of ACE-DEM in the participants (Wrobel et al. 2022). The difference between the measured points and modelled outcomes ranged 0.8–1.6 fold from modelled outputs (Fig. 6).

### Reverse dosimetry

Three reverse dosimetry approaches were evaluated using the PBK model, the results of which are introduced below and summarized in Table 3.

**Table 3:** Reverse dosimetry estimates compared to administered dose for the three different methods. The fold-deviation was calculated by dividing the calculated dose with the administered dose

Volunteer ID (Wrobel et al.)	Administered dose (mg)	Calculated dose in mg (fold deviation)		
		Method 1 (F_ue_)	Method 2 (C_24_)	Method 3 (A_24_)
*V1*	1.98	5.22 (2.64)	1.35 (0.68)	4.95 (2.50)
*V2*	1.96	7.22 (3.68)	3.08 (1.57)	2.82 (1.44)
*V3*	1.96	4.49 (2.29)	0.72 (0.37)	6.44 (3.29)
Average deviation		2.9	0.9	2.4

All calculated doses fell below a threefold deviation from the administered dose. Of all methods, the commonly used approach with the F_ue_ (method 1) and method 3, which used the cumulative amount excreted to derive a linear regression, showed the biggest difference between calculated and administered dose, with an overestimation for most of the calculated doses. Method 2, which used modelled urinary excretion and modelled dose to derive a linear regression equation, showed the smallest difference but underestimated the dose for two of the three participants.

## Discussion

In this study, we generated kinetic data from a human volunteer study and used these to develop a PBK model to estimate exposure. We presented a methodology for the back-calculation of exposure from measured ACE and ACE-DEM levels in urine. Our methods show a set-up in which volunteer studies can be used to derive kinetic parameters for other pesticides and estimate concentrations in blood and urine for the purpose of PBK-model development or validation.

While human volunteer studies by itself are not new and have been done before, including blood and feces as well as urine provides a more complete picture of the kinetics of ACE and ACE-DEM in humans as opposed to using only urine (Harada et al. 2016; Wrobel et al. 2022). Moreover, due to the availability of data from an existing human dosing experiment, we were able to verify three different approaches for reverse dosimetry.

### Oral absorption and elimination kinetics

Oral absorption was rapid, with the C_max_ for ACE reached within 1 h after dosing. The detection of ~ 1% of the dose in the feces suggests that absorption from the gastrointestinal tract was nearly complete. In addition, 1.2% of the administered dose was excreted as ACE-DEM in feces, which may indicate either microbiological conversion of ACE to ACE-DEM, or exposure to ACE-DEM from other sources or routes (EFSA, 2024). However, as the participants in this study were asked to follow an organic diet and no ACE-DEM was detected at baseline for all but one participant, the latter seems unlikely. Another possibility for excretion of ACE-DEM in the feces is via the bile. Bile-cannulated rat studies found that this pathway is not related to ACE excretion, but it could be possible that this is the case for the metabolite.

ACE concentrations decreased below the detection limit within 24 h after exposure, while ACE-DEM was detected above the detection limit up to 96 h after exposure. This indicates a fast metabolism from ACE to ACE-DEM based on high ACE-DEM levels in blood plasma and urine. These findings align with the rat data used for the approval of ACE (EFSA, 2015).

As for blood kinetics, in rat studies the T_max_ was 0.5–7 h after a single oral dose, with 96% absorption. However, the T_1/2_ of ACE in plasma for humans was 2.8 h, compared to 5.9–15 h in rats. The difference in T_1/2_ for the parent suggests an interspecies difference with regard to the metabolism of ACE. This is supported by the difference in result for the excretion in urine, where in rat studies the majority was excreted within 24 h in urine, but in humans the excretion is prolonged until after 96 h after dosing. This raises the question whether rat data would overestimate the excretion of ACE and if the current interspecies extrapolation factor of 4 is sufficient to cover the difference in kinetics between rat and human. In the original assessment, no T_1/2_ was calculated for ACE-DEM, so it is unclear whether the T_1/2_ of 51 h was similar to data from animal studies.

The prolonged excretion of ACE-DEM could be related to the ability of ACE and ACE-DEM to bind to plasma proteins. In vitro measured unbound fractions of 38 and 42% were used in the model to predict the free fraction available for metabolic processes (Taira et al. 2025). In this study, it was suggested that ACE, due to its higher protein binding affinity and lipophilicity, has the potential to accumulate in human tissues and cause toxic effects slowly. A binding of ACE to plasma proteins could be a reason for the fast observed decline in plasma levels of ACE, which could be related to protein binding instead of clearance. As such, the clearance values obtained needed to be further refined.

Earlier determined excreted fractions in urine were in line with the findings in this study. Wrobel et al. (2022) found an F_ue_ of 0.25 – 0.74% for ACE and 27 – 39% for ACE-DEM at 24 h after exposure. This is comparable to our volunteer study, which found an excreted fraction of 0.02–2.6% for ACE and 7.9–21% for ACE-DEM. The variability in these results can be related to normal variability in urinary excretion a population which ranges between 6.1 and 8.2 L/h (Van Haarst et al. 2004). A difference with the results shown by Wrobel et al. is the background exposure that occurred in the Wrobel study participants. This was corrected for in the calculation of the F_ue_s which makes them comparable with those in our study.

### Dermal absorption and elimination kinetics

For a chemical to penetrate the stratum corneum, the uppermost layer of the skin, a compound needs to have both hydrophilic and lipophilic properties and have a small molecular weight. Based on this, it was expected that ACE would readily penetrate the skin with a water solubility of 4.2 g/L (at 25 °C). When comparing the logP of ACE (1.4) with nicotine (1.2), a compound known for its dermal absorption properties, it can be expected that ACE will be readily absorbed by the skin. In our study, 13–30% of the administered dose of 0.1 mg was recovered in urine within 24 h post-exposure. However, baseline samples contained traces of the oral exposure and actual recovery might be lower. After dermal exposure, urinary concentrations of ACE and ACE-DEM were just above LOQ. All blood samples except one contained levels of ACE < LOD, and no ACE was detected in the feces.

Dermal absorption of ACE of 20–23% was reported in a porcine ex-vivo skin model using a 20% ACE formulation (Mospilan) after 1 and 24 h of exposure (Beránková et al. 2017). These values are comparable to the urinary recovery observed in our study, suggesting that ACE can be absorbed through the skin. In a risk assessment of the US Environmental Protection Agency, a dermal absorption factor of 30% based on monkey and rat studies was used (McNeilly 2009)(McNeilly 2009). Considering that dermal absorption may be overestimated in our study, actual dermal absorption under occupational conditions is likely lower than suggested by in vitro assays and animal data.

ACE can be metabolized into different metabolites, such as ACE-DEM, but also 6-CNA, N-acetyl-acetamiprid and N-acetyl-dm-acetamiprid have been identified as breakdown products in in vitro studies (Khidkhan et al. 2021). However, ACE-DEM is considered to be the main metabolite (EFSA, 2024; Ford & Casida 2006; Harada et al. 2016; Wrobel et al. 2023; EFSA, 2024; Ford & Casida 2006; Harada et al. 2016; Wrobel et al. 2023). In biomonitoring studies, ACE-DEM is often used as exposure biomarker for acetamiprid exposure, since this metabolite is specific for ACE. Yet, the metabolism of ACE to ACE-DEM and further metabolites is not characterized sufficiently, therefore it is unclear whether interaction with other chemical exposures or medication can be expected or whether individual characteristics such as age or gender play a role.

### Model performance

The PBK-model was calibrated using data from our volunteer study, specifically the time course of ACE plasma concentrations, as these showed less variability among participants. After calibration, the model was applied to data from the study by Wrobel et al. for validation. Calibration of the K_a_ and clearance values was justified based on the knowledge gaps in the mechanisms of absorption and metabolism involved in ACE kinetics. The calculated goodness of fit statistics of the model indicated that the fit of the ACE-DEM urinary concentrations is less precise compared to ACE in plasma. This can be explained by the large variation in variables that impact urinary excretion, reflected in the observed variability in the urinary excretion in the human volunteers. Different factors can potentially influence urinary excretion, such as age, gender, and fluid intake and day/night voiding (Van Haarst et al. 2004). Nevertheless, the calibrated model was able to predict the concentrations in the Wrobel et al. (2022) dataset within a margin of twofold accuracy. This indicates that even without the addition of variation in parameters, it was possible to find reasonable predictions of urinary concentrations.

The interindividual variability in metabolism presents both a limitation and an important consideration for model application. The small sample size, age, gender, or genetic factors may be important sources of variability. The population variability of these parameters should be incorporated into future model refinements.

### Reverse dosimetry validation and biomonitoring applications

Reverse dosimetry is a method commonly applied to provide risk estimates for populations. Several methods are available to calculate a dose from an intake. To the best of our knowledge this is the first study to compare model-derived doses with actual administered doses of a pesticide.

The three methods presented here all predicted the exposure within 3.5-fold accuracy. The method that used the morning urine concentration combined with the PBK-model showed to be the most accurate. This validation provides confidence that in application of the model, accurate doses can be calculated. However, we were also able to verify that other currently existing methods can predict within 3.5-fold of the actually measured dose. It is important to note that variability in urine production can influence the reliability of reverse dosimetry on spot samples. This is illustrated by earlier research which found that spot samples produced dose estimates up to 50% higher than those calculated over a full day (Aylward et al. 2017). By incorporating PBK-models into reverse dosimetry, this variability can be accounted for by specifying a range of values for the lower and upper bounds of key parameters. Method 2, which used the concentration 24 h post-exposure was most accurate, but underpredicted the doses. If a more conservative approach is desirable, method 3 which uses the total amount excreted in urine over 24 h can be preferred. When designing biomonitoring studies aimed at estimating intake and risk, this can indicate that 24 h urine sampling would be preferred. Alternatively, estimating an Fue using the PBK-model can provide opportunities to conservatively estimate risk using spot samples.

Moreover, by comparing the data of the current controlled exposure study with the earlier results from Wrobel et al. (2022), we were able to determine the background level of ACE for participants from the latter study. As dietary intake is expected to be the main exposure route for the general population, we assumed a constant daily exposure. Based on the PBK model, daily exposure at 25% of the ADI over 31 days resulted in a urinary excretion of ACE-DEM comparable to the baseline levels reported by Wrobel et al. (2022).

### Limitations and uncertainties

Estimating the dose from urinary concentrations can be challenging, as often the time lag after exposure is unknown. However, exposure estimates are important when performing risk assessments, as these create opportunities to make a comparisons with established safety levels such as the ADI. Information on exposure routes and time since exposure is not always available. When modelling an hourly concentration in blood or urine, as presented here, assumptions need to be made to back-calculate to intake. In this study, we focused on morning-urine concentrations under a constant exposure of 25% of the ADI for 31 days, reflecting common practice in biomonitoring that typically use first-morning urine. The results showed that, under this assumption, the calculated doses were still within an acceptable range of the administered doses.

With four included participants, the sample size of the current human volunteer study is very small; a larger sample size is needed to characterize population variability. Despite this, the data proved sufficient to calibrate the model and derive kinetic parameters. For example, if the CYP-isoenzymemediating ACE demethylation were known and demonstrated genetic polymorphisms, genotyping participants for that isoform might have clarified the variability in observed kinetics. In future applications, the model could be expanded to include variability of additional physiological or toxicokinetic parameters if known, for example by using Monte Carlo simulations within a probabilistic framework rather than the more deterministic approach applied here to include variability in organ size, organ blood flow, or urine production. Some background exposure was observed for the dermal exposure scenario, likely as a carry-over effect after oral administration. However, when this was taken into account, absorption through the skin remained at maximally 30% of the dose compared to 80–90% of the dose after oral exposure.

The potential for inhalation exposure to ACE has been investigated before (Marín et al. 2004). The current study design did not permit investigation of inhalation exposure because of the high acute health risks associated with inhaling the compound (Gorguner & Akgun 2010). However, based on its vapor pressure of < 1 × 10^–6^ Pa at 25 °C, ACEACE is not expected to remain in the gas phase, and exposure through air in a non-worker situation is not expected. Inhalation for workers could be incorporated in the model once sufficient inhalation-exposure data become available. Similarly, the model can be adapted for a wider scope of applications since it already includes a selection of target organs which would make it suitable for forward dosimetry applications and estimate target organ concentrations.

Human volunteer studies can provide accurate estimates of concentrations in human matrices with which PBK models can be parameterized. However, conducting such studies for a large number of pesticides is not feasible due to their laborious nature and ethical considerations. High-throughput in vitro methods capable of estimating metabolic clearance, renal clearance, and plasma-protein binding, could provide a viable alternative when human in vivo data are not available. In this paper, human volunteer studies were used to fill the data gap regarding kinetics of ACE and ACE-DEM to model reverse dosimetry. Future research should focus on comparing human data-based models with models developed using in silico and in vitro to identify the strengths and limitations of these new approach methodologies. This can help move forward to the parametrization of PBK-models without human or animal data.

## Conclusion

In conclusion, our findings demonstrate that the developed PBK model describes the kinetics of ACEACE and its main metabolite in humans within an acceptable range. The model can be applied for reverse dosimetry to support interpretation of biomonitoring data in exposure and risk assessment. Modeling was further used to evaluate different reverse-dosimetry approaches, with 24-h urine collections providing the most reliable estimates. The methodology presented here can also be extended to other chemicals, and improved knowledge of metabolism and excretion kinetics in humans is expected to further improve model reliability.

## Supplementary Information

Below is the link to the electronic supplementary material.

Supplementary Material 1

## Data Availability

Data from the human volunteer study are available upon request. Model code and scripts are available from: 10.5281/zenodo.18631991.

## Abbreviations

ACE: Acetamiprid
ACE-DEM: Acetamiprid-N-Desmethyl
ADI: Acceptable daily intake
AmFo: Ammonium formate
ARfD: Acute reference dose
AUC: Area under curve
CL_metabolic_: Hepatic clearance
CL_renal_: Renal clearance
C_max_: Maximum concentration
CYP450: Cytochrome P450
ED: Estimated dose
EFSA: European Food Safety Authority
F_ue_: Fraction excreted in urine
FA: Formic acid
HBGVs: Health-based guidance values
ICRP: International commission of radiological protection
LC–MS/MS: Liquid chromatography tandem mass spectrometry
LOD: Limit of detection
LOQ: Limit of quantification
MeOH: Methanol
nAChR: Nicotinic acetylcholine receptor
ODE: Ordinal differential equations
OECD: Organisation for economic co-operation and development
PBK: Physiologically based kinetic
PPP: Plant-protection product
QuEChERS: Quick, Easy, Cheap, Effective, Rugged, and Safe
T_max_: Time to maximum concentration
V_24_: 24 Hour urine volume
WFSR: Wageningen food and safety research
WMO: Medical research involving human subjects act / wet Medisch Onderzoek
