# Supplementary material for: Integrated human toxicokinetics of acetamiprid using urine, blood, and feces data in physiologically-based kinetic modelling for reverse dosimetry
Source: Arch Toxicol. 2026 May 25;100(9):3961–76. doi: 10.1007/s00204-026-04459-z (PMC13453981; doi:10.1007/s00204-026-04459-z)
Supplement: Supplementary file 1 [file 204_2026_4459_MOESM1_ESM.docx]

# Supplementary materials

Contents

[Supplementary materials	1](#_Toc222389889)

[S1: Questionnaire	2](#_Toc222389890)

[S2: Overview of sampling and scheduling	4](#_Toc222389891)

[S3: QC for LC/MS-MS	5](#_Toc222389892)

[S4: PBK model parameters	7](#_Toc222389893)

[S5: Derivation of clearance parameters	10](#_Toc222389894)

[S6: Sensitivity analysis with Morris and eFAST	11](#_Toc222389895)

[S7: Model validation	22](#_Toc222389896)

[S8: Model fit before and after calibration	25](#_Toc222389897)

## S1: Questionnaire

**Have you had any pets in the past month? (Poultry/livestock not included.)**
Options: Yes / No → If no, continue with question 9.

**Which pets did you have in the past month? Please also indicate the number of animals per type.**
Options: Dog (number), Cat (number), Rodent (number), Bird (number), Other (number).

**Did you have direct (skin) contact with poultry/livestock or other animals besides pets in the past month?**
Options: Yes / No.
If yes: specify which animals you were in contact with.

**Have you used tobacco products and/or an e-cigarette in the past month?**
Options: Yes / No → If no, continue with question 12.

**Which tobacco products and/or e-cigarette did you use in the past month? Please also indicate the amount per day. (Multiple answers possible.)**
Options: Cigarettes (per day), Roll-your-own tobacco (per day), Cigarettes (duplicate in original text), E-cigarette (per day), Other (per day).

**How would you describe your living environment?**
Options:
– Urban – in or on the edge of a large city
– Urban – in or on the edge of a village
– Rural – outside a village or city, close to agricultural land
– Rural – living directly on or within agricultural land

**In the past month, have you eaten vegetables, fruit and/or herbs from your (kitchen) garden at home?**
Options: Yes / No

**Have you used prescription-only medicines in the past month?**
Options: Yes / No → If no, continue with question 16.

**Which prescription-only medicines did you use in the past month? (If you do not remember the name, indicate what the medicine was for.)**
Open text.

**Have you used medicines and/or homeopathic products in the past month that are available without a prescription (e.g., at the supermarket, drugstore or pharmacy)? (For example paracetamol, cough syrup, vitamin pills, nicotine patches.)**
Options: Yes / No → If no, continue with question 18.

**Which non-prescription medicines and/or homeopathic products did you use in the past month? (If you do not remember the name, indicate what the medicine was for.)**
Open text.

**For each category, indicate whether you used any of the products below in the past month. (Use for work does not count.)**
Categories with Yes/No:
– Products against fleas and ticks in pets
– Products against head lice
– Products against insects (lice, ants, caterpillars, mosquitoes, etc.)
– Products against weeds
– Products against fungi
– Products against slugs
– Products against rats and mice (not mousetraps)
– Other products (specify)

**Which pesticides and/or biocides did you use in and around your home in the past month?**
Instructions: Provide the product name and the Ctgb authorization number. If you do not remember the name, indicate what you used it for (e.g., “against aphids,” “against weeds”).

**If you have any additional information that you think may be important for the measurements in your blood, stool, urine, and skin, please note it below.**
Open text.

## S2: Overview of sampling and scheduling

**Figure S1.1:** An overview of sampling as performed in the human volunteer study. Blood samples were collected at 0, 0.5, 1, 1.5, 2, 2.5, 3, 4, 5, 6, 8 and 10 h after dosing.

## S3: QC for LC/MS-MS

**Blood plasma**

n = 6 calibration points (0.125 - 10 ng/mL); LOQ of 0.125 ng/mL with a <20 RSD%.

| Compound | Method | LOD | LOQ | QC initial validation | | | | | | | | | On-going validation (QCs concurrently analysed with the samples) | | | | |
| --- | --- | --- | --- | --- | --- | --- | --- | --- | --- | --- | --- | --- | --- | --- | --- | --- | --- |
|  |  |  |  | **level-1: 0.1 ng/mL** | | | **level-2: 0.5 ng/mL** | | | **level-3: 2.5 ng/mL** | | | **level-2: 2.5 ng/mL** | | |  |  |
|  |  | **ng/mL** | | **Rec (ave)** | **RSDr** | **N** | **Rec (ave)** | **RSDr** | **N** | **Rec (ave)** | **RSDr** | **N** | **Rec (ave)** | **RSDwR** | **N** | **Remarks** |  |
| Acetamiprid | 1 LC-MS/MS pos | 0.001 | 0.125 | 97.4 | 3.37 | 6 | 108.1 | 3.8 | 6 | 97 | 3.74 | 4 | 106.7 | 10.4 | 43 | L1=0,125, L2=1, L3=10 ng/ml |  |
| Acetamiprid-N-desmethyl | 1 LC-MS/MS pos | 0.012 | 0.125 | 91.2 | 20 | 6 | 106.1 | 5.1 | 6 | 96.8 | 2.8 | 4 | 103.7 | 11.6 | 43 | L1=0,125, L2=1, L3=10 ng/ml |  |

**Feces: recoveries for QC samples concurrently analysed with the study samples**

| **Spike level to feces:** | **Batch 1** | | **Batch 2** | | **Batch 3** | |
| --- | --- | --- | --- | --- | --- | --- |
|  | **1 µg/kg** | **2.5 µg/kg** | **1 µg/kg** | **2.5 µg/kg** | **1 µg/kg** | **2.5 µg/kg** |
|  | Recovery in % | | | | | |
| Acetamiprid | 82 | 96 | 92 | 96 | 103 | 101 |
| Acetamiprid-N-desmethyl | 106 | 109 | 91 | 92 | 107 | 99 |
|  |  |  |  |  |  |  |
|  |  |  |  |  |  |  |
|  |  |  |  |  |  |  |

**Urine : recoveries for QC samples concurrently analysed with the study samples**

| **Spike level in urine:** | **Batch 1** | | **Batch 2** | |  |  |
| --- | --- | --- | --- | --- | --- | --- |
|  | **0.1 ng/mL** | **0.5 ng/mL** | **0.1 ng/mL** | **0.5 ng/mL** |  |  |
|  | Recovery in % | | | |  |  |
| Acetamiprid | 111 | 105 | 109 | 94 |  |  |
| Acetamiprid-N-desmethyl | 117 | 110 | 100 | 106 |  |  |

## S4: PBK model parameters

**Table S3.1:** Overview of all parameters and units used in the PBK model. Tissue:plasma partitioning coefficients were calculated using Rodgers and Rowlands’ formula. Absorption rate constant and fraction absorbed were fitted to data from all four participants from the human volunteer study. Estimation of clearance values is elaborated on in **Supplementary S2**.

| **Parameter** | **Value** | **Unit** | **Source** |
| --- | --- | --- | --- |
| Weight (body) | 70 | kg | (ICRP, 2002) |
| Dose (90% ADI) | 0.023 | mg/kg bw/day | (EFSA (European Food Safety Authority) et al., 2024) |
| **Kinetic parameters** | | | |
| Absorption rate constant | 15.0 | h^-1^ | Fitted |
| Fraction absorbed (gut) | 0.80 | Fraction | Fitted |
| Fraction unbound in plasma, ACE | 0.58 | Fraction | (Taira et al., 2025) |
| Fraction unbound in plasma, ACE-DEM | 0.62 | Fraction | (Taira et al., 2025) |
| Renal clearance, ACE | 0.30 | L/hr | Fitted |
| Renal clearance, ACE-DEM | 1.20 | L/hr | Fitted |
| Metabolic clearance, ACE | 43.0 | L/hr | Fitted |
| Metabolic clearance, ACE-DEM | 1.00 | L/hr | Fitted |
| Urine production rate | 0.70 | L/hr | (Van Haarst et al., 2004) |

| **Physicochemical properties** | | | |
| --- | --- | --- | --- |
| ***ACE*** | | | |
| Molecular weight | 222.68 | g/mol | PubMed |
| LogP | 1.4 | Unitless | PubMed |
| pKa | 0.7 | Unitless | PubMed |
| Blood:plasma partitioning | 1.0 | Unitless | Assumption |
| ***ACE-DEM*** | | | |
| Molecular weight | 208.65 | g/mol | PubMed |
| LogP | 1.3 | Unitless | PubMed |
| pKa | 0.7 | Unitless | PubMed |
| Blood:plasma partitioning | 1.0 | Unitless | Assumption |

| **Tissue:plasma partitioning coefficients** | | |
| --- | --- | --- |
| **Tissue** | **ACE** | **ACE-DEM** |
| Adipose | 0.89 | 1.22 |
| Bone | 1.73 | 1.86 |
| Brain | 1.89 | 2.09 |
| Gut | 1.56 | 1.74 |
| Heart | 0.89 | 1.06 |
| Kidney | 1.08 | 1.26 |
| Liver | 1.38 | 1.56 |
| Lung | 0.72 | 0.90 |
| Muscle | 1.06 | 1.24 |
| Spleen | 1.09 | 1.28 |
| Skin | 1.14 | 1.31 |

* In absence of experimental data blood:plasma partitioning was assumed to be 1.

## S5: Derivation of clearance parameters

Clearance values were derived combining urine and blood plasma data from the same volunteer. The total apparent clearance (CL_total_, µg/L) was calculated using equation 1 (Eq. 1), where f*dose represents the dose administered corrected for the assumed bioavailability, and AUC the area under the curve of the acetamiprid plasma concentration over 24 hours (in µg/L/h). Renal clearance (CL_renal_, µg/L) was estimated according to equation 2 (Eq. 2), where A_e_ denotes the total amount excreted in the urine in 24 hours.

${CL}_{total}=\frac{f*dose}{A{UC}_{0-24}}$	*[Eq. 1]*

${CL}_{renal}=\frac{A_{e}}{A{UC}_{0-24}}$	*[Eq. 2]*

By subtracting CL_renal_ from CL_total_, the metabolic clearance (CL_metabolic_, µg/L) of the parent could be estimated. (Eq. 3).

${CL}_{metabolic}= {CL}_{total}-{CL}_{renal}$	*[Eq.3]*

## S6: Sensitivity analysis with Morris and eFAST

**Figure S6.1:** Outcomes from Morris’ test and eFAST sensitivity indices. Abbreviations: CL_Ki_M = Kidney clearance ACE-DEM; CL_Me_M = Metabolic clearance ACE-DEM; CL_Ki = Kidney clearance, ACE; CL_Me = Metabolic clearance; BP = blood:plasma partitionin, ACE; BPm = blood:plasma partitioning, ACE-DEM

| Parameter | Unit | mu | mu_star | sigma |
| --- | --- | --- | --- | --- |
| BP | - | 188.999 | 188.99903 | 70.46872 |
| WEIGHT | Kg | -41.1377 | 41.137662 | 20.24047 |
| Qurine | L/H | -38.0224 | 38.022437 | 14.9004 |
| fup | Fraction | 34.50681 | 34.506807 | 13.90151 |
| CL_Me | L/H | 34.30928 | 34.309278 | 14.32034 |
| CL_Ki_M | L/H | 3.572328 | 8.8457337 | 9.650915 |
| fupm | Fraction | 2.976223 | 8.7283793 | 9.777714 |
| CL_Me_M | L/H | -0.82252 | 0.8225244 | 0.611724 |
| CL_Ki | L/H | -0.54896 | 0.5489559 | 0.262507 |
| ka | /h | 0.113263 | 0.1132629 | 0.058191 |
| QC | L/H | 0 | 0 | 0 |
| Fabs | Fraction | 0 | 0 | 0 |
| BPm |  | 0 | 0 | 0 |

**Table 6.1:** Results from the Morris’ test

| Parameter |  | Distribution | First_Order | Total_Effect |
| --- | --- | --- | --- | --- |
| BP | - | uniform | 0.821858357 | 0.844975757 |
| WEIGHT | Kg | lognormal | 0.059067828 | 0.073901229 |
| CL_Me | L/H | lognormal | 0.042827545 | 0.052065531 |
| Qurine | L/H | uniform | 0.031364811 | 0.039481176 |
| fup | Fraction | uniform | 0.02598409 | 0.033320439 |
| CL_Ki_M | L/H | lognormal | 0.000632442 | 0.007441792 |
| fupm | Fraction | uniform | 0.000269643 | 0.006431155 |
| CL_Me_M | L/H | lognormal | 2.80E-05 | 0.005176916 |
| CL_Ki | L/H | lognormal | 1.32E-05 | 0.004954092 |
| Fabs | Fraction | uniform | 5.64E-07 | 0.004941365 |
| BPm | - | uniform | 8.63E-07 | 0.004915659 |
| ka | /h | uniform | 1.40E-07 | 0.004830268 |
| QC | L/H | lognormal | 2.30E-07 | 0.004738278 |

**Table 6.2**: Results from the eFAST

| Parameter |  | Current_Value | Morris_Distribution | Morris_Range | eFAST_Distribution | eFAST_Range |
| --- | --- | --- | --- | --- | --- | --- |
| QC | L/H | 390 | Uniform | [351.0000, 429.0000] | lognormal | [351.0000, 429.0000] |
| WEIGHT | Kg | 70 | Uniform | [63.0000, 77.0000] | lognormal | [63.0000, 77.0000] |
| ka | /h | 15 | Uniform | [13.5000, 16.5000] | uniform | [13.5000, 16.5000] |
| fup | Fraction | 0.579 | Uniform | [0.5211, 0.6369] | uniform | [0.5211, 0.6369] |
| fupm | Fraction | 0.623 | Uniform | [0.5607, 0.6853] | uniform | [0.5607, 0.6853] |
| Qurine | L/H | 0.06 | Uniform | [0.0540, 0.0660] | uniform | [0.0540, 0.0660] |
| Fabs | Fraction | 0.8 | Uniform | [0.7200, 0.8800] | uniform | [0.7200, 0.8800] |
| CL_Ki | L/H | 0.3 | Uniform | [0.2700, 0.3300] | lognormal | [0.2700, 0.3300] |
| CL_Ki_M | L/H | 43 | Uniform | [38.7000, 47.3000] | lognormal | [38.7000, 47.3000] |
| CL_Me | L/H | 1.2 | Uniform | [1.0800, 1.3200] | lognormal | [1.0800, 1.3200] |
| CL_Me_M | L/H | 1 | Uniform | [0.9000, 1.1000] | lognormal | [0.9000, 1.1000] |
| BP | - | 1 | Uniform | [0.9000, 1.1000] | uniform | [0.9000, 1.1000] |
| BPm | - | 1 | Uniform | [0.9000, 1.1000] | uniform | [0.9000, 1.1000] |

**Table 6.3:** Parameter distributions used for Morris and eFAST

| Parameter |  | mu | mu_star | sigma | Distribution | First_Order | Total_Effect |
| --- | --- | --- | --- | --- | --- | --- | --- |
| BP | - | 188.999 | 188.99903 | 70.46872 | uniform | 0.821858357 | 0.844975757 |
| WEIGHT | Kg | -41.1377 | 41.137662 | 20.24047 | lognormal | 0.059067828 | 0.073901229 |
| CL_Me | L/H | 34.30928 | 34.309278 | 14.32034 | lognormal | 0.042827545 | 0.052065531 |
| Qurine | L/H | -38.0224 | 38.022437 | 14.9004 | uniform | 0.031364811 | 0.039481176 |
| fup | Fraction | 34.50681 | 34.506807 | 13.90151 | uniform | 0.02598409 | 0.033320439 |
| CL_Ki_M | L/H | 3.572328 | 8.8457337 | 9.650915 | lognormal | 0.000632442 | 0.007441792 |
| fupm | Fraction | 2.976223 | 8.7283793 | 9.777714 | uniform | 0.000269643 | 0.006431155 |
| CL_Me_M | L/H | -0.82252 | 0.8225244 | 0.611724 | lognormal | 2.80E-05 | 0.005176916 |
| CL_Ki | L/H | -0.54896 | 0.5489559 | 0.262507 | lognormal | 1.32E-05 | 0.004954092 |
| Fabs | Fraction | 0 | 0 | 0 | uniform | 5.64E-07 | 0.004941365 |
| BPm | - | 0 | 0 | 0 | uniform | 8.63E-07 | 0.004915659 |
| ka | /h | 0.113263 | 0.1132629 | 0.058191 | uniform | 1.40E-07 | 0.004830268 |
| QC | L/H | 0 | 0 | 0 | lognormal | 2.30E-07 | 0.004738278 |

**Table 6.4:** Results from the total sensitivity ranking

**R-Code**

*# ===========================================================================*

*library(mrgsolve)*

*library(sensitivity)*

*library(ggplot2)*

*library(dplyr)*

*library(tidyr)*

*# ===========================================================================*

*# STEP 1: LOAD MRGSOLVE MODEL*

*# ===========================================================================*

*# Load your model*

*model_dev*

*# ===========================================================================*

*# STEP 2: DEFINE PARAMETERS AND DISTRIBUTIONS*

*# ===========================================================================*

*# parameter definitions*

*params <- c('QC', 'WEIGHT', 'ka', 'fup', 'fupm', 'Qurine', 'Fabs',*

*'CL_Ki', 'CL_Ki_M', 'CL_Re', 'CL_Re_M', 'BP', 'BPm')*

*cur.value <- c(390, 70, 15, 0.579, 0.623, 0.06, 0.8,*

*0.3, 43, 1.2, 1, 1, 1)*

*distributions <- c('lognormal', 'lognormal', 'uniform', 'uniform', 'uniform',*

*'uniform', 'uniform', 'lognormal', 'lognormal', 'lognormal',*

*'lognormal', 'uniform', 'uniform')*

*# ===========================================================================*

*# STEP 3: CREATE DISTRIBUTION SPECIFICATIONS*

*# ===========================================================================*

*# MORRIS: Uniform distribution for ALL parameters (±10% of current value)*

*morris_distributions <- list()*

*for (i in 1:length(params)) {*

*morris_distributions[[params[i]]] <- list(*

*type = "uniform",*

*min = cur.value[i] * 0.9,*

*max = cur.value[i] * 1.1,*

*current = cur.value[i]*

*)*

*}*

*# eFAST: Lognormal for physiological/clearance, Uniform for others (±10%)*

*efast_distributions <- list()*

*for (i in 1:length(params)) {*

*if (distributions[i] == "lognormal") {*

*# For lognormal: set median = current value*

*# sdlog controls variability (0.1 gives ~±10% range within 1 SD)*

*efast_distributions[[params[i]]] <- list(*

*type = "lognormal",*

*meanlog = log(cur.value[i]),*

*sdlog = 0.105, # This gives approximately ±10% range at 1 SD*

*min = cur.value[i] * 0.9,*

*max = cur.value[i] * 1.1,*

*current = cur.value[i]*

*)*

*} else { # uniform*

*efast_distributions[[params[i]]] <- list(*

*type = "uniform",*

*min = cur.value[i] * 0.9,*

*max = cur.value[i] * 1.1,*

*current = cur.value[i]*

*)*

*}*

*}*

*# Print distribution summary*

*cat("\n=== MORRIS DISTRIBUTIONS (All Uniform ±10%) ===\n")*

*for (param in params) {*

*dist <- morris_distributions[[param]]*

*cat(sprintf("%s: [%.4f, %.4f] (current: %.4f)\n",*

*param, dist$min, dist$max, dist$current))*

*}*

*cat("\n=== eFAST DISTRIBUTIONS ===\n")*

*for (i in 1:length(params)) {*

*param <- params[i]*

*dist <- efast_distributions[[param]]*

*cat(sprintf("%s (%s): ", param, distributions[i]))*

*if (dist$type == "uniform") {*

*cat(sprintf("[%.4f, %.4f]\n", dist$min, dist$max))*

*} else {*

*cat(sprintf("median=%.4f, sdlog=%.3f, range=[%.4f, %.4f]\n",*

*exp(dist$meanlog), dist$sdlog, dist$min, dist$max))*

*}*

*}*

*# ===========================================================================*

*# STEP 4: SAMPLING FUNCTIONS*

*# ===========================================================================*

*# Function to convert quantiles to parameter values for eFAST*

*quantile_to_param_efast <- function(param_name, quantiles) {*

*dist <- efast_distributions[[param_name]]*

*if (dist$type == "uniform") {*

*values <- qunif(quantiles, dist$min, dist$max)*

*} else if (dist$type == "lognormal") {*

*values <- qlnorm(quantiles, dist$meanlog, dist$sdlog)*

*values <- pmax(pmin(values, dist$max), dist$min) # Truncate*

*}*

*return(values)*

*}*

*# Function to convert quantiles to parameter values for Morris*

*quantile_to_param_morris <- function(param_name, quantiles) {*

*dist <- morris_distributions[[param_name]]*

*values <- qunif(quantiles, dist$min, dist$max)*

*return(values)*

*}*

*# ===========================================================================*

*# STEP 5: CREATE WRAPPER FUNCTION FOR MRGSOLVE MODEL*

*# ===========================================================================*

*run_pbk_model <- function(param_values) {*

*# Create parameter list*

*param_list <- setNames(as.list(param_values), params)*

*# Update model with new params*

*mod_updated <- param(modDev, param_list)*

*# Define dosing*

*dose_event <- ev(amt = 100, cmt = 1)*

*# Run simulation*

*out <- mrgsim(mod_updated, events = dose_event, end = 24, delta = 0.1)*

*# Compute AUC of mCurine*

*output <- sum(diff(out$time) * (head(out$mCurine, -1) + tail(out$mCurine, -1)) / 2)*

*return(output)*

*}*

*# Test the wrapper function*

*test_output <- run_pbk_model(cur.value)*

*cat("\n=== Model Test ===\n")*

*cat("Test model run output with current values:", test_output, "\n")*

*# ===========================================================================*

*# STEP 6: MORRIS SCREENING (Uniform ±10% for all parameters)*

*# ===========================================================================*

*cat("\n=== Running Morris Screening Analysis ===\n")*

*cat("Using UNIFORM distribution (±10%) for ALL parameters\n\n")*

*# Morris method parameters*

*r <- 100 # Number of repetitions (50-200 recommended)*

*levels <- 6 # Number of levels (4-10 recommended)*

*# Create Morris design in [0,1] space*

*morris_design <- morris(model = NULL,*

*factors = params,*

*r = r,*

*design = list(type = "oat", levels = levels, grid.jump = 3),*

*binf = rep(0, length(params)),*

*bsup = rep(1, length(params)))*

*# Transform [0,1] samples to actual parameter space (all uniform ±10%)*

*morris_X_transformed <- morris_design$X*

*for (i in 1:ncol(morris_X_transformed)) {*

*morris_X_transformed[, i] <- quantile_to_param_morris(params[i],*

*morris_design$X[, i])*

*}*

*# Run model for all Morris samples*

*cat("Running", nrow(morris_X_transformed), "model evaluations for Morris...\n")*

*morris_results <- numeric(nrow(morris_X_transformed))*

*pb <- txtProgressBar(min = 0, max = nrow(morris_X_transformed), style = 3)*

*for (i in 1:nrow(morris_X_transformed)) {*

*morris_results[i] <- run_pbk_model(morris_X_transformed[i, ])*

*if (i %% 50 == 0) setTxtProgressBar(pb, i)*

*}*

*close(pb)*

*# Process Morris results*

*tell(morris_design, morris_results)*

*# Plot Morris results*

*png("morris_results.png", width = 800, height = 600)*

*plot(morris_design, main = "Morris Screening: Elementary Effects (Uniform ±10%)")*

*dev.off()*

*# Create summary table*

*morris_summary <- data.frame(*

*Parameter = params,*

*mu = apply(morris_design$ee, 2, mean),*

*mu_star = colMeans(abs(morris_design$ee)),*

*sigma = apply(morris_design$ee, 2, sd)*

*)*

*morris_summary <- morris_summary[order(-morris_summary$mu_star), ]*

*cat("\n=== Morris Summary (ranked by importance) ===\n")*

*print(morris_summary, row.names = FALSE)*

*# ===========================================================================*

*# STEP 7: eFAST ANALYSIS (Lognormal for physiol/clearance, Uniform for others)*

*# ===========================================================================*

*cat("\n=== Running eFAST Global Sensitivity Analysis ===\n")*

*cat("Using LOGNORMAL for physiological/clearance, UNIFORM for others\n\n")*

*# eFAST parameters*

*n <- 1000 # Sample size per search curve (500-2000 recommended)*

*M <- 4	# Interference parameter (usually 4)*

*# Create quantile function specification for eFAST*

*# q must be a VECTOR of character strings (not a list!)*

*# q.arg must be a list of lists*

*q <- character(length(params))*

*q.arg <- list()*

*for (i in 1:length(params)) {*

*param <- params[i]*

*dist <- efast_distributions[[param]]*

*if (dist$type == "uniform") {*

*q[i] <- "qunif"*

*q.arg[[param]] <- list(min = dist$min, max = dist$max)*

*} else if (dist$type == "lognormal") {*

*q[i] <- "qlnorm"*

*q.arg[[param]] <- list(meanlog = dist$meanlog, sdlog = dist$sdlog)*

*}*

*}*

*# Create eFAST design with proper distributions*

*efast_design <- fast99(model = NULL,*

*factors = params,*

*n = n,*

*M = M,*

*q = q,*

*q.arg = q.arg)*

*# Apply truncation to lognormal parameters (post-sampling)*

*for (i in 1:length(params)) {*

*if (distributions[i] == "lognormal") {*

*param <- params[i]*

*dist <- efast_distributions[[param]]*

*efast_design$X[, param] <- pmax(efast_design$X[, param], dist$min)*

*efast_design$X[, param] <- pmin(efast_design$X[, param], dist$max)*

*}*

*}*

*# Run model for all eFAST samples*

*cat("Running", nrow(efast_design$X), "model evaluations for eFAST...\n")*

*efast_results <- numeric(nrow(efast_design$X))*

*pb <- txtProgressBar(min = 0, max = nrow(efast_design$X), style = 3)*

*for (i in 1:nrow(efast_design$X)) {*

*efast_results[i] <- run_pbk_model(efast_design$X[i, ])*

*if (i %% 500 == 0) setTxtProgressBar(pb, i)*

*}*

*close(pb)*

*# Process eFAST results*

*tell(efast_design, efast_results)*

*# Print eFAST results*

*cat("\n=== eFAST Results ===\n")*

*print(efast_design)*

*# Create summary table*

*efast_summary <- data.frame(*

*Parameter = params,*

*Distribution = distributions,*

*First_Order = efast_design$D1 / efast_design$V,*

*Total_Effect = 1 - (efast_design$Dt / efast_design$V)*

*)*

*efast_summary <- efast_summary[order(-efast_summary$Total_Effect), ]*

*cat("\n=== eFAST Summary (ranked by Total Effect) ===\n")*

*print(efast_summary, row.names = FALSE)*

*# ===========================================================================*

*# STEP 8: VISUALIZATION*

*# ===========================================================================*

*# Morris plot - mu* vs sigma*

*morris_plot_data <- morris_summary*

*p1 <- ggplot(morris_plot_data, aes(x = mu_star, y = sigma)) +*

*geom_point(size = 4, color = "steelblue") +*

*geom_text(aes(label = Parameter), hjust = -0.2, vjust = -0.2, size = 3.5) +*

*labs(title = "Morris Screening Results (Uniform ±10%)",*

*x = "μ* (Overall Influence)",*

*y = "σ (Non-linearity/Interactions)") +*

*theme_minimal() +*

*theme(plot.title = element_text(hjust = 0.5, size = 14, face = "bold"))*

*ggsave("morris_scatter.png", width = 10, height = 7)*

*print(p1)*

*# eFAST bar plot*

*efast_plot_data <- efast_summary %>%*

*pivot_longer(cols = c(First_Order, Total_Effect),*

*names_to = "Index_Type",*

*values_to = "Sensitivity_Index")*

*p2 <- ggplot(efast_plot_data, aes(x = reorder(Parameter, Sensitivity_Index),*

*y = Sensitivity_Index,*

*fill = Index_Type)) +*

*geom_bar(stat = "identity", position = "dodge") +*

*coord_flip() +*

*scale_fill_manual(values = c("First_Order" = "skyblue",*

*"Total_Effect" = "coral"),*

*labels = c("First Order (Main Effect)",*

*"Total Effect (Main + Interactions)")) +*

*labs(title = "eFAST Sensitivity Indices",*

*x = "Parameter",*

*y = "Sensitivity Index",*

*fill = "") +*

*theme_minimal() +*

*theme(plot.title = element_text(hjust = 0.5, size = 14, face = "bold"),*

*legend.position = "bottom")*

*ggsave("efast_barplot.png", width = 10, height = 7)*

*print(p2)*

*# Combined plot showing distribution type*

*efast_plot_data_dist <- efast_summary %>%*

*pivot_longer(cols = c(First_Order, Total_Effect),*

*names_to = "Index_Type",*

*values_to = "Sensitivity_Index")*

*p3 <- ggplot(efast_plot_data_dist,*

*aes(x = reorder(Parameter, Sensitivity_Index),*

*y = Sensitivity_Index,*

*fill = Index_Type)) +*

*geom_bar(stat = "identity", position = "dodge") +*

*coord_flip() +*

*facet_grid(Distribution ~ ., scales = "free_y", space = "free_y") +*

*scale_fill_manual(values = c("First_Order" = "skyblue",*

*"Total_Effect" = "coral"),*

*labels = c("First Order", "Total Effect")) +*

*labs(title = "eFAST Sensitivity Indices by Distribution Type",*

*x = "Parameter",*

*y = "Sensitivity Index",*

*fill = "") +*

*theme_minimal() +*

*theme(plot.title = element_text(hjust = 0.5, size = 14, face = "bold"),*

*legend.position = "bottom",*

*strip.text = element_text(face = "bold"))*

*ggsave("efast_by_distribution.png", width = 10, height = 8)*

*print(p3)*

*# ===========================================================================*

*# STEP 9: EXPORT RESULTS FOR PAPER*

*# ===========================================================================*

*# Parameter summary table*

*param_summary <- data.frame(*

*Parameter = params,*

*Current_Value = cur.value,*

*Morris_Distribution = "Uniform",*

*Morris_Range = sprintf("[%.4f, %.4f]",*

*cur.value * 0.9,*

*cur.value * 1.1),*

*eFAST_Distribution = distributions,*

*eFAST_Range = sprintf("[%.4f, %.4f]",*

*cur.value * 0.9,*

*cur.value * 1.1)*

*)*

*write.csv(param_summary, "parameter_distributions.csv", row.names = FALSE)*

*# Combined sensitivity results*

*combined_results <- merge(morris_summary, efast_summary, by = "Parameter")*

*combined_results <- combined_results[order(-combined_results$Total_Effect), ]*

*write.csv(combined_results, "sensitivity_results.csv", row.names = FALSE)*

*# Separate tables for Morris and eFAST*

*write.csv(morris_summary, "morris_results.csv", row.names = FALSE)*

*write.csv(efast_summary, "efast_results.csv", row.names = FALSE)*

*cat("\n=== Analysis Complete ===\n")*

*cat("Results saved:\n")*

*cat(" - parameter_distributions.csv (Methods section table)\n")*

*cat(" - morris_results.csv\n")*

*cat(" - morris_scatter.png\n")*

*cat(" - efast_results.csv\n")*

*cat(" - efast_barplot.png\n")*

*cat(" - efast_by_distribution.png\n")*

*cat(" - sensitivity_results.csv (combined results)\n")*

*# ===========================================================================*

*# INTERPRETATION GUIDE*

*# ===========================================================================*

*cat("\n=== HOW TO INTERPRET RESULTS ===\n\n")*

*cat("MORRIS SCREENING (Uniform ±10% for all parameters):\n")*

*cat(" μ* (mu_star): Overall importance - higher = more influential\n")*

*cat(" σ (sigma): Non-linearity or interaction effects\n")*

*cat(" High μ*, Low σ: Linear, important parameter\n")*

*cat(" High μ*, High σ: Non-linear or strong interactions\n")*

*cat(" Low μ*: Parameter has little influence on output\n\n")*

*cat("eFAST INDICES (Mixed distributions):\n")*

*cat(" First Order Index (S): Variance due to parameter alone\n")*

*cat(" Total Effect Index (ST): Main effect + all interactions\n")*

*cat(" ST - S: Variance due to interactions with other parameters\n")*

*cat(" ST > 0.1: Parameter is influential (common threshold)\n")*

*cat(" Sum of S indices ≈ 1: Model is mostly additive\n")*

*cat(" Sum of S indices << 1: Strong parameter interactions\n\n")*

*cat("DISTRIBUTION RATIONALE:\n")*

*cat(" Uniform: Used for all parameters in Morris (screening phase)\n")*

*cat(" Lognormal in eFAST: Physiological parameters (QC, WEIGHT) and\n")*

*cat("	clearances (CL_Ki, CL_Ki_M, CL_Re, CL_Re_M)\n")*

*cat(" Uniform in eFAST: Fractions and partition coefficients\n")*

*cat("	(ka, fup, fupm, Qurine, Fabs, BP, BPm)\n\n")*

*# Print top 5 most influential parameters*

*cat("TOP 5 MOST INFLUENTIAL PARAMETERS (by eFAST Total Effect):\n")*

*top5 <- head(efast_summary[order(-efast_summary$Total_Effect), ], 5)*

*for (i in 1:nrow(top5)) {*

*cat(sprintf(" %d. %s (ST = %.3f, %s distribution)\n",*

*i, top5$Parameter[i], top5$Total_Effect[i], top5$Distribution[i]))*

*}*

## S7: Model validation

**This section summarizes the outcomes during the model validation and adaptation of parameters to improve model fit.**

**Figure S7.1**: Effect of changing the absorbed fraction (Fabs) from the gut lumen to the gut on venous ACE concentration (in µg/L).

**Figure S7.2**: Effect of changing the metabolic (CL_Me, in L/h)) and kidney (CL_Ki, in L/h) clearance on venous ACE concentration (in µg/L).

**Figure S7.3**: Effect of changing the absorption constant (Ka, in h^-1^) on venous ACE concentration (in

µg/L).

## S8: Model fit before and after calibration

Based on the sensitivity analyses performed in Supplementary S7, parameters were adjusted to improve the model fit on the blood plasma concentration curves. In addition, a validation was done by plotting the urinary excretion of ACE-DEM over measured concentrations.

**Figure S8:** Model fit before and after parameter scaling.

## S9: Comparison of human volunteer data to Wrobel et al. (2022)

Figure S9.1: A comparison between the average measured concentrations of ACE-DEM (µg/L) over time in Wrobel et al. (2022, blue line) with the findings from the current study (red line). Shaded regions indicate range (min-max).
